# Tough and Robust Mechanically Interlocked Gel–Elastomer Hybrid Electrode for Soft Strain Gauge

**DOI:** 10.1002/advs.202301116

**Published:** 2023-05-03

**Authors:** Jianren Huang, Anbang Chen, Songjiu Han, Qirui Wu, Jundong Zhu, Jiayu Zhang, Yujia Chen, Jiantao Liu, Lunhui Guan

**Affiliations:** ^1^ CAS Key Laboratory of Design and Assembly of Functional Nanostructures Fujian Key Laboratory of Nanomaterials Fujian Institute of Research on the Structure of Matter Chinese Academy of Sciences Fuzhou 350108 China; ^2^ Xiamen Port Holding Group co. Ltd Xiamen 361012 China

**Keywords:** gel–elastomer, motion classification, robustness, soft strain gauge

## Abstract

Soft strain gauges provide a flexible and versatile alternative to traditional rigid and inextensible gauges, overcoming issues such as impedance mismatch, the limited sensing range, and fatigue/fracture. Although several materials and structural designs are used to fabricate soft strain gauges, achieving multi‐functionality for applications remains a significant challenge. Herein, a mechanically interlocked gel–elastomer hybrid material is exploited for soft strain gauge. Such a material design provides exceptional fracture energy of 59.6 kJ m^−2^ and a fatigue threshold of 3300 J m^−2^, along with impressive strength and stretchability. The hybrid material electrode possesses excellent sensing performances under both static and dynamic loading conditions. It boasts a tiny detection limit of 0.05% strain, ultrafast time resolution of 0.495 ms, and high linearity. This hybrid material electrode can accurately detect full‐range human‐related frequency vibrations ranging from 0.5 to 1000 Hz, enabling the measurement of physiological parameters. Additionally, the patterned soft strain gauge, created through lithography, demonstrates superior signal–noise rate and electromechanical robustness against deformation. By integrating a multiple‐channel device, an intelligent motion detection system is developed, which can classify six typical human body movements with the assistance of machine learning. This innovation is expected to drive advancements in wearable device technology.

## Graphical abstract

A tough and robust mechanically interlocked gel–elastomer hybrid material presents excellent strain sensing performances within static and dynamic loading. Through the lithography fabrication, the material is exploited for the fabrication of soft strain gauge, and exhibits superior signal–noise‐rate and electromechanical robustness. Then, an intelligent human motion detection system is constructed by integrating multiple channel soft strain gauge.

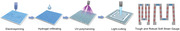

## 1 Introduction

Wearable, stretchable strain sensor technologies have immense potential in creating intelligent human‐centric devices, thanks to their compatibility with the dynamic and curved surface human skin. In particular, the emergence of soft alternatives to traditional rigid strain gauges has shown promise in wearable systems, offering excellent sensing performance over a broad response range.^[^ ^1^ ^]^ Soft strain gauges have the advantage of overcoming the limitations of impedance mismatch, low sensitivity, and the integration challenges associated with inextensible conventional metallic strain gauges in highly deformable systems.^[^ ^2^ ^]^ While researchers have explored various materials and structural designs to create soft strain gauges, achieving optimal functionality remains a significant challenge for applications involving human–machine interaction.^[^ ^3^, ^4^, ^5^, ^6^, ^7^ ^]^

First, the mechanical performance of the constituent materials is a critical factor in fabricating reliable and durable sensory devices for wearable applications.^[^ ^8^, ^9^ ^]^ Irregular shapes in patterned soft strain gauge configurations can result in stress concentration in the structure under tensile loading, which may lead to the destruction of the electronics by crack propagation.^[^ ^10^ ^]^ As a result, using sensing materials that are inherently brittle may not be feasible for manufacturing highly deformable soft strain gauges.^[^ ^11^ ^]^ A meticulous evaluation of the deformable design and inherent toughness of the sensing materials is crucial in wearable and flexible electronics, as the sensing elements must endure repeated stretching, bending, and twisting while still maintaining their functionality. Moreover, the combination of characteristics typically necessitates further material design involving conductivity, stability, and durability. Meanwhile, considering the essential criteria for extended operational life, the sensing material must not only remain its strength upon fracture but also resist crack propagation under cyclic fatigue loads.

Second, the main research trends for sensing material include enhancing the electromechanical response performance in terms of linearity, sensing limits, durability, time‐resolution, electrical robustness, and accomplishing additional functions.^[^ ^12^, ^13^, ^14^ ^]^ Among them, linear response ability (a characteristic of commercial strain gauges) is one of the most critical parameters that determine the precision of the detected signal. This parameter is desirable to decrease implementation complexity and facilitate calibration. Synchronously, the detection limits of flexible strain sensors are dependent on the specific design and application requirements.^[^ ^15^ ^]^ It is crucial to optimize the response time for each specific application to ensure accurate and reliable measurements. Besides, achieving high‐performance sensing material with high electrical stability and damage resistance against complex loading remains a significant challenge. Therefore, fabricating a soft strain gauge requires several sensing material properties to promote the maturation and widespread deployment of flexible sensor technologies.

Third, conventional flexible strain sensor configurations, such as bulk/film shape intrinsic strain sensors that are more compatible for easy fabricating, typically exhibit an unsatisfactory signal baseline, which results in a low signal–noise ratio (SNR) because of their sensitivity to extra surrounding noise.^[^ ^3^, ^16^, ^17^, ^18^ ^]^ Although a number of high‐performance strain response mechanisms have been developed, these electronics have primarily been restricted to practical applications due to the unmentioned structure design in flexible strain sensors. On the one hand, most reported flexible strain sensors are developed with anisotropic materials, which are always only sensitive to the direction that is parallel to the loading from test equipment.^[^ ^13^, ^19^, ^20^ ^]^ These strategies may imply the fabricated sensors' sense availability while without a specific direction. Similarly, traditional strain gauges with parallel grid wires composed of polyimide film and copper–nickel alloy are only sensitive to the single axial strain loading direction while being insensitive to the unconcerned other loadings.^[^ ^21^ ^]^ Actually, many practical fields require sensitivity in a specific direction with electromechanical robustness.

Another important consideration is the electromechanical robustness of the sensor, which is typically determined by its ability to tolerate a long‐term cycle number of strain loading (>1000).^[^ ^17^, ^20^, ^22^, ^23^ ^]^ However, although this may be sufficient for rigid electronics, cyclic stretching tests do not adequately capture the myriad of operating states that occur in flexible systems.^[^ ^24^ ^]^ Specifically, sensing devices are typically conformably affixed or worn on the surface of human skin, and human activities involve irregular skin deformations and joint movements that inevitably twist or bend the soft sensor.^[^ ^25^ ^]^ Therefore, soft strain sensors employed on the human body must be able to withstand large‐scale bending, twisting, and compressing deformations under regular operation and should be resistant to damage from all of these. Besides, the capability to decouple or reject mentioned off‐axis deformations can help support inherent disturbance rejection and minimize the complexity of control progress. Moreover, flexible sensing electronics working in unconstrained conditions may encounter several adverse factors during experiments and deployment, including impacts, punctures, and over‐extension, which subjected the sensor to tensile strains significantly exceeding the operating range. Consequently, robust sensory devices must function reliably when subjected to variable extra work conditions. As a result, fabrication methods must be developed to obtain unique strain gauge structures of diverse functional electronic components and form reliable devices capable of acquiring high‐quality signals.

Here, we developed a flexible sensing electrode by embedding an elastic thermoplastic polyurethanes (TPU) fibers network scaffold into another ionic gel matrix by mechanically interlocking. Such a unique structure design leads to exceptional mechanical strength (8.4 MPa) while maintaining high stretchability (1425%). It also exhibits excellent fracture energy (59.6 kJ m^−2^) and fatigue threshold (3330 J m^−2^), allowing toughness design for durability without causing failure. The prepared TPU hybrid gel electrodes can simultaneously exhibit high linearity (*R* ^2^ = 0.996) over a wide sensing range (600%) and low hysteresis. They also demonstrate other superior characteristics such as time resolution (0.495 ms), ultralow detection limit (0.05%), full‐range dynamic response ability (0.5 to 1000 Hz), and damage resistance. These outstanding properties enable electrodes to monitor critical physiological parameters with accuracy and reliability.

Inspired by a commercial strain gauge, the hybrid gel electrodes were patterned using laser‐cutting to create scalable fabrication soft strain gauge sensors. These sensors are resilient to adverse operating conditions and deliver a high signal–noise rate, as well as insensitivity to off‐axial loadings such as bending, torsion, and pressure. To demonstrate the potential application of this technology, we configured a wearable movement monitoring system by utilizing a multi‐channel soft strain gauge. Using a trained neural network model, the systems can identify six different movement states with an approximate classification precision of 100%.

## 2 Result and Discussion

**Figure 1:**
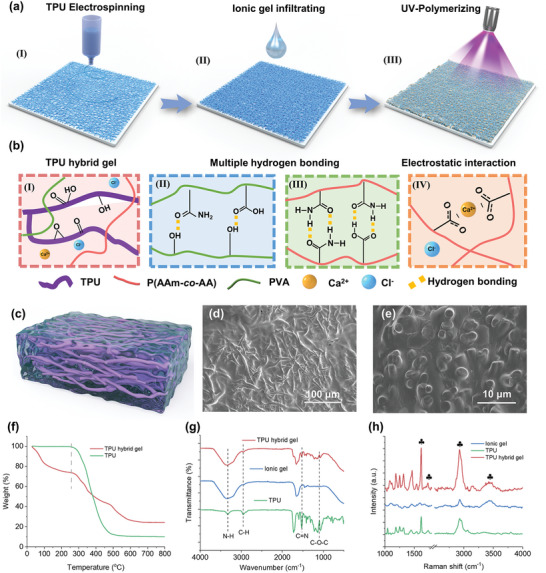
Fabrication and characterization of TPU hybrid gel. a) Schematic illustration of the fabrication processes for TPU hybrid gel and strain gauge sensor. I) Electrospinning process for integrating of TPU fiber network. II) Infiltration of hydrogel precursor into fiber network. III) In situ polymerization of the ionic gel to achieve mechanically interlocked electrodes. b) Schematic illustration of multiple interactions in the gel–elastomer hybrid composites. c) Schematic illustration of prepared TPU hybrid gel. The d) surface view and e) cross‐sectional SEM image of the TPU hybrid gel. f) TGA curve of TPU fiber network and TPU hybrid gel. g) FT‐IR patterns and h) Raman spectra of TPU fiber network, ionic gel, and TPU hybrid gel, respectively.

Flexible electrodes have traditionally utilized elastomers and polymer gels, but these materials pose challenges when attempting to integrate them into hybrid structures that maintain their individual characteristics and exhibit reliable interaction. To address this issue, interpenetrating polymer networks, which are polymers that combine multiple networks while maintaining the primary characteristics of each, have been proposed as a solution.^[^ ^26^ ^]^ Here, we developed a mechanical interlock strategy to integrate elastomers and ionic gels into hybrids (**Figure** **1**a). Specifically, the TPU substrate was freshly created by using electrospinning, which results in a structurally deformable elastic fiber web. Additionally, the electrospun TPU fiber network is softer than the bulk film, making it suitable for wearable applications on the skin. Hydrogel is a typical material composed of polymer chains that can absorb and retain large amounts of water to form a gel‐like structure and was used as the ionic gel component. The incorporated ionic salts act as a charge transport medium, providing the hydrogel with unique properties that are advantageous for flexible and wearable electronics. The polyvinyl alcohol (PVA) and polyacrylamide (AAm)/acrylic acid (AA) ionic hydrogels acted as conductive phases in composite material due to their tough and soft performance. The manufacturing process and interaction between the components are illustrated in Figure 1b and Figure S1, Supporting Information. To enhance the hydrophilicity of the TPU substrate, TPU fibers were treated with oxygen plasma to attach abundant functional groups, rendering the surface more hydrophilic and enabling better adhesion of the ionic gel matrix (Figure 1b‐I and Figure S2, Supporting Information). Then, the ionic gel precursor was synthesized by slowly evaporating the precursor solution, consisting of P(AAm‐*co*‐AA), PVA, and chloride calcium (CaCl_2_) aqueous solution, under ambient conditions until the final moisture equilibrium was reached. We developed a high‐performance ionic gel precursor by systematically adjusting the CaCl_2_ content (Figures S3 and S4, Supporting Information). An optimal 20 wt% CaCl_2_ content was achieved, resulting in an ionic gel with exhibited optimized properties, including equilibrium water content, ionic conductivity (4.38 S m^−1^), and mechanical strength (0.65 MPa@1800% strain). Subsequently, the gel precursor was infiltrated into TPU substrate in the glass model, where it filled the porous TPU webs through capillary action, forming the TPU hybrid gel after UV‐initiated polymerization. The abundant porous TPU fibers network enabled the uniform penetration of the liquid ionic gel precursor, as demonstrated in Figures S5 and S6, Supporting Information. The subsequent polymerization result in the formation of a consistent hybrid gel with a bicontinuous mechanically interlocked structure.

The polymerization of AAm and AA monomers was carried out in the presence of PVA as a molecular template. The rigid physical PVA network interpenetrated with a flexible P(Aam‐*co*‐AA) chain, facilitating the establishment of strong hydrogen bonding interactions among the amino, —OH, and —COOH functional groups, thus promoting the formation of the polymer network (Figure 1b‐II,III). Besides, the high‐concentration CaCl_2_ served as the ionic crosslinking agent, which can interact with the deprotonated —COO^−^ groups (Figure 1b‐IV).^[^ ^22^ ^]^ The presence of both covalent and ionic crosslinked networks imparted high toughness and conductivity to the TPU hybrid gel.

The elastomeric TPU fiber was utilized to produce porous films via the electrospinning method to form a substrate network. The SEM image of Figure S7a,b, Supporting Information, reveals the formation of micro‐meshes and a porous structure among the interlocked TPU fibers, with a diameter of 1–3 µm for a single fiber. Besides, they were naturally merged junctions together, which was beneficial to the mechanical performance of composites.^[^ ^27^ ^]^ The fibers demonstrated excellent elastic behavior, remaining intact even after applying a 100% tensile strain load, with the junctions remaining firmly connected (Figure S5c,d, Supporting Information). The morphology of the ionic gel and TPU hybrid gel was obtained after freeze‐drying the ionic gel and TPU as well as their uniform hybridization. The hybrid gel exhibited a mechanical interlock structure with thin layers of pure ionic gel on the outside, and no visible interlayer delamination or fiber pulling out was observed. This suggests that TPU fiber and gel were in tight contact with strong interfacial adhesion.

The resulting homogeneous mechanically interlocked structure on the sample surface provided an abundant fiber network channel for efficient charge transport (Figure 1d). Furthermore, as presented in Figure 1e, the TPU fibers in the hybrid gel retained their initial structure well, interlocked ionic gel. The corresponding EDS mapping images also clearly showed the presence of Ca^2+^ and Cl^−^ ions (Figure S8 and Table S1, Supporting Information), confirming the successful locking of ionic gel into the TPU substrate network. Additionally, the ionic gel content in TPU hybrid gel was quantitated by TGA analysis in Figure 1f. The weight change demonstrated that ≈30% of the hybrid structure was composed of the infiltrated ionic gel. Fourier Transform Infrared (FT‐IR) and Raman spectra were used to characterize the functional groups of samples and the interactions between various chemical groups, as shown in Figure 1g,h. The characteristic peaks at ≈3335 and 1099 cm^−1^ observed in the neat TPU nanofiber were attributed to the stretching vibrations of the —OH and —NH functional groups, respectively.^[^ ^28^ ^]^ Notably, the peak at 3335 cm^−1^ was presented in the TPU polymer due to its hydrophilic nature. Moreover, the peak at 2960 cm^−1^ in FT‐IR spectra was assigned to the stretching vibrations of the —CH bonds in the alkene functional group. The shift of the C=O and N—H vibration peaks in the TPU hybrid gel toward lower wavelengths compared to neat TPU suggested the possibility of hydrogen bonding between the functional groups of the ionic gel and the C=O and N—H groups. In Figure 1h, Raman spectra were employed to confirm the presence of ionic gel elements within the TPU hybrid gel. In detail, TPU‐enhanced ionic gel exhibited characteristic peaks that may be attributed to the interfacial force transfer between TPU and the gel chain. Hydrogen bonding between the functional groups of the two components can lead to a more significant interaction and hence better electromechanical properties, improved load transfer during dynamic tensile deformation, and enhanced mechanical performance. This was because the hydrogen bonds can act as “interfacial bridges” between the two components, improving the interfacial adhesion and resulting in more efficient load transfer between the two materials. As a consequence, the TPU hybrid gel can exhibit superior mechanical stability and performance compared to neat TPU.^[^ ^29^ ^]^

**Figure 2:**
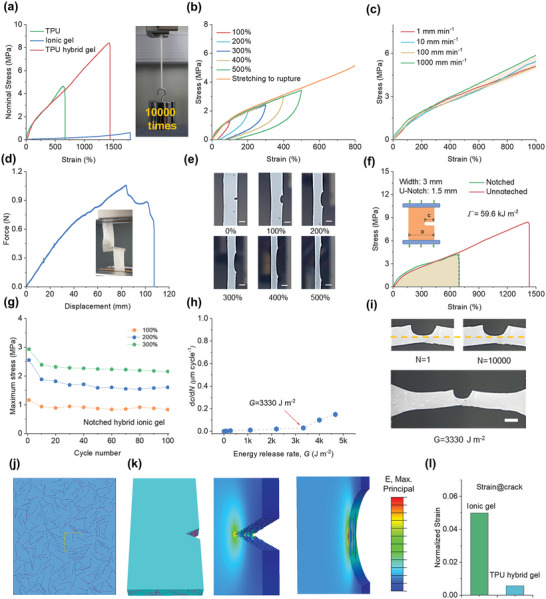
Mechanical performance of TPU hybrid gel. a) Stress–strain curves of the TPU, ionic gel, and hybrid gel under monotonic loading. The insert demonstrated that a lightweight TPU hybrid gel film weighing only 0.1 g was able to bear a weight of 1 kg. b) Cyclic stress–strain curves of TPU hybrid gel at increasing strains from 100% to 500% and rupture. c) Tensile stress–strain curves of TPU hybrid gel at different strain rates. d) Trouser tearing experiments on the TPU hybrid gel. e) Photographs of the notched hybrid gel stretched to different strains. f) Typical stress–strain curves of the unnotched and notched TPU hybrid gel. g) The maximum stress of the notched sample during the cyclic stretching test. h) Crack extension per cycle d*c/*d*N* versus applied energy release rate *G* for TPU hybrid gel. i) Photographs of the notched sample with applied *G* from 1 to 10 000 cycle. j) Computational model of TPU hybrid gel. k) FEA model with an initial crack. k) The FEA simulates strain distribution in the crack propagation area when the samples apply 100% elongation. l) The result shows the normalized strain distribution of TPU hybrid gel and ionic gel.

To engineer flexible electronics, it is crucial to consider the mechanical properties of foundation materials. As shown in **Figure** **2**a, the ionic gel can undergo uniaxial tension up to 18 times its initial dimension, despite possessing a low tensile strength of 0.62 MPa. Notably, the mechanical performance of electrospun TPU‐enhanced hybrid gel was distinct from pure TPU fiber film and ionic gel due to the mechanically interlocked mixture. This hybrid structure revealed mechanical strength and toughness, with elastic moduli calculated from the original linear segment in stress–strain curves at 1.5 MPa, comparable to the moduli of many biological tissues.^[^ ^30^ ^]^ Moreover, an increase in the elongation of hybrid gel was discovered in the tensile stress–strain curve. This phenomenon can be attributed to the fact that, during the stretching process, the soft polymer chain of the ionic gel in the hybrid was the first to rupture. As the material was loaded, the force‐bearing component of TPU hybrid gel gradually changes from an ionic gel matrix to a highly stressed and aligned TPU fiber network. This shift in the load‐bearing component enabled the material to dissipate a significant amount of the fracture energy, resulting in greater resistance to stress‐induced breaking or failure. The highly stretchable hybrid gel can bear loads ≈10 000 times heavier than its own weight without experiencing structural damage (Figure 2a insert and Video S1, Supporting Information). The remarkable and apparent transition from resilience to damping has been extensively acknowledged in load‐bearing tissues as a protective mechanism against accidental impact.^[^ ^31^ ^]^ Indeed, the TPU enhanced hybrid gel illustrated high stretchability (1425%) and mechanical strength (8.4 MPa) during stretching. By combining the characteristics of TPU and ionic gel, the resulting material exhibited high rigidity and considerable ductility. These features made TPU hybrid gel a desirable candidate for soft bioelectronics applications that involve significant deformations, including wearable devices and flexible electronic sensors.

Figure 2b shows the results of loading–unloading tests on the same TPU hybrid gel sample under varied strains without resting intervals. The TPU fiber network in the hybrid possessed both elasticity and plasticity, making the hybrid gel highly elastic with almost identical loading curves to the single tensile curve to break. This phenomenon was attributed to the failure of covalent bonds in the polymer webs, which resulted in a substantial reduction in strength during the consecutive releasing process. Furthermore, successive stretching cycles of the TPU hybrid gel exhibited a distinct hysteresis in the first stretching cycle, but this hysteresis loop did not occur in the subsequent stretching processes, suggesting significant energy dissipation through dynamic interchain crosslinks (Figure S9, Supporting Information). After undergoing 100 loading/unloading cycles at a 300% strain, the hysteresis loops of the TPU hybrid gel were almost overlapped, providing strong evidence for good repeatability. The desirable elastic behavior of the hybrid gel can reduce the hysteresis of the output electromechanical response signals. More critically, in contrast, to hydrogel elastomers proposed previously, the stress–strain curves of the TPU hybrid gel exhibited negligible dependency on stretching speed, which coincides with human skin with more accuracy and further identified the considerable role of TPU fiber network in controlling the deformation (Figure 2c).

In general, gel materials face fundamental challenges when it comes to mechanical damage resistance. Fortunately, the composite structure of the TPU hybrid gel, consisting of both rigid fiber networks and flexible hydrogel polymer networks, endows the material with the high tear, fracture, and fatigue resistances. Trouser tearing tests were performed to assess the strength and durability of fabric or material by measuring the force required to tear it.^[^ ^32^, ^33^ ^]^ As shown in Figure 2d, the force–displacement curves illustrated that the TPU hybrid gel possessed high tearing strength and energy. The tearing process showed that the sample was significantly deformed over an area in the crack tip with a maximum dimension of a few millimeters, indicating that both the matrix and fibers dissipate energy. The low‐viscosity precursor solution of the TPU hybrid gel permeated the TPU fiber network entirely during preparation, constructing mechanically interlocking networks after polymerization in addition to a stable interface. The interface can effectively transfer the stress by shear deformation of the gel matrix.

Thanks to its remarkable energy dissipation capability, the pre‐damaged TPU hybrid gel exhibited great crack tolerance. The single‐notch strategy involves creating a single notch or groove in the TPU hybrid and subjecting it to various types of loading or stress to determine its resistance to crack initiation and propagation, following the well‐established Greensmith method.^[^ ^34^ ^]^ It was noteworthy that the hybrid gel with circular pre‐cut cracks could be stretched to approximately seven times its original dimension even when the pre‐cut cracks were 50% of the sample's length (Figure 2e). The stretching process of the pre‐crack TPU hybrid gel was illustrated in Video S2, Supporting Information, which showed that the TPU hybrid gel display an extraordinary crack‐inhibition behavior in that the pre‐crack progressively expanded along the longitudinal direction but nearly did not advance into hybrid gel even at large strains until the occurrence of the sample rupture. Figure 2f exemplified the stress–strain response of the notched samples, exhibiting exceptional crack resistance during the tearing procedure with a rupture strain of 695%, and a fracture energy (*Γ*) of 59.6 kJ m^−2^ was obtained. Additionally, as indicated in Figure S10, Supporting Information, the pre‐crack TPU hybrid gel specimen with a notch width of 0.75 mm (1/4 of length) can be stretched to more than ten times its initial length, achieving an ultimate stress of 4.9 MPa at the rupture strain of 1034.7%. The fracture energy was calculated as 51.5 kJ m^−2^. In contrast, much lower fracture energy of 1.51 and 25.1 kJ m^−2^ were observed for the ionic gel sample and neat TPU fiber film, respectively (Figure S11, Supporting Information).

The notched specimens with various original shapes, including linear and circular incisions, were subjected to uniaxial stretching. As demonstrated in Figure S12, Supporting Information, all the samples with different shapes of pre‐notched did not exhibit crack propagation under monotonic tensile deformation, even for the linear pre‐cut incision (1/3 of the samples dimension) with obvious stress concentration. The sample can be elongated to six times its initial length. The TPU hybrid gel owes its crack tolerance to two main factors. First, the embedded TPU fiber network produced a considerable force transfer effect through the large elastomer/gel modulus ratios, which created a significant energy dissipation area and thus prevented cracks from propagating further.^[^ ^32^ ^]^ Sequentially, due to the mechanical interlocking, TPU fibers in the energy dissipation regime were broken instead of being pulled out by debonding, which substantially increased the elastic energy that the fibers can store and dissipate, endowing the TPU hybrid gel with extraordinary fracture energy.

The performance of flexible devices is often limited by their resistance to fatigue and fracture under repeated cyclic loading (evaluated by fracture threshold), rather than their tensile strength and toughness under a monotonic load (evaluated by fracture energy).^[^ ^35^ ^]^ Despite the dissipation mechanisms, hybrid gel materials reported thus far still exhibit fatigue fractures under long‐term cyclic loading, since the energy required to fracture a single layer of polymer chains remains largely unaffected by extra dissipation behaviors.^[^ ^36^ ^]^ Most load‐bearing elastomers, such as rubber and silicone, possess high toughness but are also prone to fatigue and have a lower fracture threshold value. As a result, they can endure significant deformation without breaking but are prone to fail under repeated stress over time.^[^ ^37^ ^]^ To assess the fatigue resistance of the TPU hybrid gel, repeated loads with various tensile strains were performed on the pre‐notched specimens (Experimental Section and Figure S13, Supporting Information). The mechanical strength of the notched ionic gel was substantially reduced before damage at a maximum strain of 200%, and cracks developed throughout the whole sample within hundreds of cycles (Figure S14, Supporting Information). However, TPU hybrid gel exhibited the ability to endure 10 000 successive stretching and releasing cycles without any noticeable crack growth (Video S3, Supporting Information).

Shakedown is a phenomenon that can alter the stress–strain response of a material and reduce the likelihood of fracture under repeated loading.^[^ ^38^ ^]^ As illustrated in Figure 2g and Figure S15, Supporting Information, most of the energy dissipation and strength softening occurred during the initial stretching–releasing cycle. While energy dissipation led to stress relaxation in the TPU hybrid gel, the samples showed almost no crack propagation and maintained similar mechanical performance during the subsequent loadings cycles. The notched TPU hybrid gel maintained high stress levels even during long‐term loading, indicating a robust hybrid structure with high resistance to static fatigue damage caused by consecutive cyclic mechanical loading.

The fatigue threshold of a material is defined as the minimum cyclic stress or strain that it can endure without significant degradation or failure over time. This property is essential in applications where materials are subjected to repetitive loading.^[^ ^37^, ^39^ ^]^ Detailed calculation methods can be found in the Supporting Information. We linearly extrapolated the data in the plane of energy release reate(G) and Crack extension per loading cycle (d*c/*d*N)*, and calculated the intercept of the *G* axis as the threshold of the TPU hybrid gel, which was found to be 3330 J m^−2^ (Figure 2h). We further employed corresponding *G* to the pre‐crack specimen over 10 000 cycles of loading and found no crack propagation (Figure 2i). In contrast, the threshold fatigue of ionic gel was orders of magnitude lower than that of TPU hybrid gel (Figure S16, Supporting Information). For comparison, we included some mechanical parameters of hydrogels and elastomers reported in the literature in Figure S17 and Table S2, Supporting Information. Apparently, our TPU hybrid gel demonstrated remarkable fatigue and fracture resistance.

To investigate the toughening mechanism and fracture behavior of TPU hybrid gel, we conducted finite element analysis (FEA) simulations to simulate crack evolution during stretching. According to the SEM image, We constructed representative volume element model of random TPU fibers and ionic gel using the ABAQUS secondary development interfaces. As illustrated in Figure 2j, the TPU fibers were embedded into the ionic gel matrix, creating a mechanically interlocked structure. During loading, the TPU fiber network was elongated into long and thin fibers, similar to the way steel bars are incorporated into cement to form a tough structure in reinforced concrete. Figure 2k and Figure S18, Supporting Information, demonstrated the FEA model and corresponding Von Mises stress distribution of the TPU hybrid gel and ionic gel. Under strain loading, the ionic gel exhibited an evident stress concentration in the crack area, causing the crack rapidly propagate horizontally and leading to structural failure. In contrast, due to the high rigidity of the TPU fibers, significant stress concentration was transferred to the TPU network. As demonstrated in the strain gradient program result of Figure 2l, the ionic gel exhibited greater elongation than TPU hybrid gel under the same deformation loading. The effective deflection and rude behaviors during pre‐notched TPU hybrid gel stretching endowed a typical sideway crack inhibition, reducing the local stress concentration around the crack tip region and endowing the samples with high fracture toughness, thus preventing crack propagation.

Besides, the anti‐dehydrating and anti‐freezing properties of the TPU hybrid gel were also characterized to demonstrate its stability. As calculated from Nyquist plots in Figure S19a,b, Supporting Information, the conductivity of hybrid gel remained at 1.61 S m^−1^ after storage at −20 °C, which was close to the value of 1.83 S m^−1^at room temperature. As revealed in Figure S19c, Supporting Information, the samples exhibited excellent anti‐freezing ability, which could maintain as high as 7.51 MPa strength at 1077% tensile strain at −20 °C. These results demonstrated that the water molecules in the hybrid gel were firmly fixed by CaCl_2_, inhibiting the formation of the crystal lattice or preventing water evaporation, resulting in satisfactory electromechanical properties of the TPU hybrid gel at a low temperature. As illustrated in Figure S20, Supporting Information, the samples maintained steady ions conductivity and mechanical performance during long‐term storage. The high ionic hydration of the dissolved CaCl_2_ implied strong bond interaction between cation/anion–water molecule pairs, resulting in greater difficulty of water evaporation. In various environmental cases, no obvious signs of material failure of the hybrid gel were observed, and the sample almost remained constant performance.

**Figure 3:**
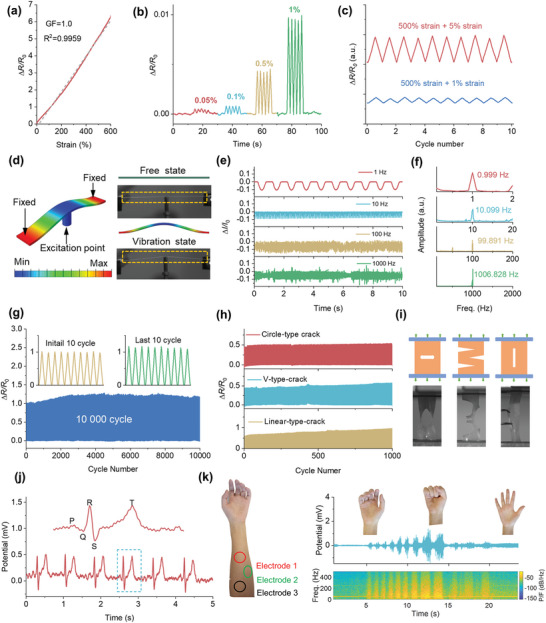
Electromechanical response performance of the TPU hybrid gel electrode. a) Relative electrical resistance–strain response curves of the TPU hybrid gel electrode. b) Resistance response of electrode for ultra‐low strain detection. c) Resistance responses of an electrode by superimposing 1% and 5% strain on fixed 500% tensile strain. d) Images and schematic illustrations of vibration generation and dynamic detection system. e) Resistance changes of TPU hybrid gel electrode under different vibration frequencies. f) Corresponding FFT‐processed frequency spectrum. g) The relative resistance responses of the electrode during 10 000 stretching and releasing cycles at strain rates of 100%. h) Cyclic performance of the notched TPU hybrid gel electrode at a strain of 50%. i) The photographs demonstrating the durability of the notched electrode. j) The ECG signals detected by the TPU hybrid gel electrodes. The enlarged view shows the P, Q, R, S, and T waves. k) EMG signals collected in different gesture and their energy spectrum.

The combination of mobile ions and excellent elasticity in hybrid ionic gel confers the material with the unique ability to sense strain and modulate its electrical resistance in response to applied pressure or deformation. As a result, it has gained considerable attention as a promising material for developing stretchable and flexible sensors for various applications. **Figure** **3**a demonstrates that the related resistance change of the TPU hybrid gel electrode substantially increased with the applied loading, typical for stretchable ionic sensing material. The gauge factor (GF) of the TPU hybrid gel electrode was determined to be 1.0 at a sensing range from 0 to 600%.

This statement suggests that the sensitivity of a device or system was sufficient for a wide range of sensing applications, and can effectively detect changes or inputs in various environments. The relationship between changes in resistance and strain was accurately represented by a straight line, with a high *R*‐squared value of 0.996. This high *R*‐square value indicated that the data points fit the linear relationship very well, making calibration and measurement straightforward in a specific application. Moreover, as shown in Figures S21 and S22, Supporting Information, the corresponding stretching–releasing cycle of resistance change demonstrated ultra‐low hysteresis and high linearity within a border operating range. It should also be noted that the electrodes demonstrated fast and linear response, and short recovery time and do not exhibit electrical hysteresis during the stretching test, which were desirable characteristics for functional sensors (Figure S23, Supporting Information).

Figures S24 and S25, Supporting Information, indicated the time‐resolved electrical response of an electrode subjected to gradient static loading stimuli. The TPU hybrid gel electrode could distinguish stepwise loading by a 0.5% strain. The electrodes displayed steady signal response and a high SNR, enabling real‐time deformation monitoring. Subsequently, the electrical signal response of the electrode was evaluated under an ultralow strain to determine its behavior as a sensor. A mechanical testing stage equipped with an ultrasensitive force cell was employed to apply subtle deformation stimuli to the electrode, and the electrical signal response was measured. As demonstrated in Figure 3b, cyclic sensing tests toward ultralow tensile strains of 0.05%, 0.1%, 0.5%, and 1% were conducted. The TPU hybrid gel electrode produced stable electrical signals even at an extremely low strain of 0.05%, demonstrating its high sensitivity. Traditional strain sensors face challenges in detecting both large and subtle strains simultaneously. However, the TPU hybrid gel electrode can effectively capture subtle strains even under extremely high tensile strains (500%). Figure 3c demonstrates the electrode's ultrahigh operating sensory resolution, capable of detecting slight strains of 1% and 5% even at 500% tensile strain. Furthermore, as shown in Figure S26, Supporting Information, the electrode also exhibited reliable electromechanical response under superimposing tiny strains (1%) on stretched fixed strains (100%, 200%, 300%, and 500%)

The relative resistance of the electrode maintained relatively constant with increasing tensile speed, indicating that the stretching rate did not significantly affect the electrode and rendering it suitable for real‐world applications (Figure S27, Supporting Information). However, detecting tiny, high‐frequency vibrations generated by dynamic loading has historically been challenging.^[^ ^40^ ^]^ To evaluate the performance of the TPU hybrid gel electrode in detecting dynamic stimulation, we configured a vibration measurement and control system (detail in the Supporting Information and illustrated in Video S4, Supporting Information). Figure 3d illustrates the relationship between vibration and loading during testing. The shaker generated sustained vibrations via the signal generator. In the experiments, the electrode was subjected to base excitation at frequencies ranging from 0.5 to 1000 Hz to demonstrate its ability to precisely monitor high‐frequency dynamic loading changes. The electrodes were found to possess high sensitivity in detecting mechanical vibrations over a broad frequency range (Figure 3e and Figure S28, Supporting Information). Meanwhile, the acquired current–time response showed a similar sinusoidal wave with good quality even for a vibration frequency of 1000 Hz. Applying FFT to the electrode signal, frequency components present in the signal were obtained and represented in the form of a frequency spectrum,^[^ ^16^ ^]^ and presented in Figure 3f. The frequencies extracted from the frequency spectrum for the base excitation frequencies of 1, 10, 100, and 1000 Hz were 0.999, 10.099, 99.891, and 1006.828 Hz, respectively, in exact agreement with the input base excitation frequencies. This demonstrated that the TPU hybrid gel electrode preciously measures the excitation frequency with an error of only 0.5%. It can detect vibrations across a wide range of frequencies, making it a valuable tool for measuring and analyzing various types of mechanical vibrations. Additionally, the electrode's high sensitivity to subtle changes in strain and its quick response time contributed to its high frequency‐sensing resolution. As illustrated in Figure S29, Supporting Information, the electrode achieved an ultrafast response and recovery time of ≈0.495 ms during the high frequency dynamic loading, real response times may be even quicker than what has been recorded, taking into account the contact duration between the static force and electronics during tests. Table S3, Supporting Information, compared the TPU hybrid gel electrode with other recent available reports and showed that it possessed one of the lowest detection limits and the fastest response time. The TPU hybrid gel electrode was capable of sensing the vibration frequency ranging from 0.5 to 1000 Hz, which included most human neural receptor touch frequencies.^[^ ^40^ ^]^

The TPU hybrid gel electrode was subjected to repeated stretching and releasing of 100% tensile strain for over 10 000 cycles to evaluate its stability (Figure 3g). The cyclic test results indicated that the TPU hybrid gel electrode was stable and durable, as it showed a rapid and reliable response to applied loading and returned to its initial state after each cycle of loading and unloading. Traditional sensor devices are susceptible to structural damage, which can lead to a loss of integrity and equipment failure. To exemplify the effect of mechanical destruction on electromechanical performance, some typical cracks were artificially incorporated into TPU hybrid gel electrode. Figure S30, Supporting Information, illustrates the contrasting durability of the notched TPU hybrid gel and ionic gel electrode. The ionic gel was incapable of preventing crack propagation under continuous cyclic loading, leading to a relatively short service life (less than 100 s). Figure 3h,i presents the real‐time resistance variation for notched TPU hybrid gel electrodes with three different pre‐cut cracks. The prepared electrode exhibited stable electrical signal output for over 1000 cyclic tests as the subsequent sample loading. Remarkably, the TPU hybrid gel electrode continued to operate even when various notch demonstrated its high intrinsic fracture and fatigue resistance (Video S5 and Figure S31, Supporting Information). The TPU hybrid electrode demonstrated excellent robustness in tolerating structural failure while maintaining reliable electromechanical properties.

The high deformability of TPU hybrid gel electrode allowed conformal integration of the electronics on the dynamically curved surfaces of the skin. It is conceivable that the inherent porous of electrodes improve the breathability during long‐term attaching to the skin.^[^ ^10^ ^]^ Moreover, we further investigated the biocompatibility of the TPU hybrid gel electrode by observing the skin's reaction after attaching the electrode for different durations. There was no skin irritation after applying the prepared TPU hybrid gel electrodes for 1 h (Figure S32, Supporting Information). As demonstrated in Figure S33, Supporting Information, the electrodes showed low interfacial impedance, indicating conformal contact with skin. The multifunctional electrode built‐in TPU hybrid gel could characterize the various physiological information from the skin. For example, electrodes were placed on the arms and legs to measure the electrocardiogram (ECG) recordings. The left and right arm electrodes were used to measure the electrical potential between them, while the leg electrode serves as a reference point, producing a clear and accurate ECG signal. The ECG signals captured by the sensor exhibited details, and all of the P, Q, R, S, and T waves can be clearly distinguished, and these signals represented different phases of the heart's electrical cycle and can be used to determine heart rate,^[^ ^41^, ^42^ ^]^ monitor for arrhythmias, and diagnose other cardiac conditions (Figure 3j and Video S6, Supporting Information). The heart rate was also captured from the ECG signal through a Fourier transform. Figure S34, Supporting Information, demonstrates that the frequency of heart rate was 1.4 Hz, corresponding to 81 times per minute. Similarly, in the EMG measurement, the TPU hybrid gel electrodes placed on the upper arm were used to measure the electrical potential generated by the muscle fibers during contraction. The reference electrode on the palm helped to eliminate electrical noise and provided a reference point. Three target hand gestures were induced and corresponding EMG signals from the data acquisition system were recorded by prepared electrodes. As demonstrated in Figure 3k and Video S7, Supporting Information, the electrodes acquired the distinct waves arising from the contraction of various muscle groups under grasping conditions. The EMG for grasping had the highest average energy, while natural states had the lowest. Therefore, the power spectral density of the EMG was calculated as the signal features. This spatiotemporal information can be helpful in the diagnosis of a range of conditions, such as muscle weakness, nerve damage, and muscle disorders.^[^ ^8^ ^]^

Soft strain gauges are an emerging technology that offers new capabilities in soft machines and are made of materials that possess a combination of elasticity, toughness, high linear sensitivity, and low hysteresis.^[^ ^23^ ^]^ These soft strain gauges can provide more accurate and reliable strain measurements and can be facilely tailored in terms of dimension and shape to meet the requirements of flexible electronics using novel manufacturing technologies. To take advantage of the strain‐depended resistive sensory mechanism, we have fabricated prototypical flexible strain sensors with a specific arrangement of conductive elements that enhance the sensor's response to load in a specific direction. The conductive electrode was patterned into the shape of a meander curve, with the mechanically interlocked gel–elastomer hybrid electrode serving as the sensing element (method in the Experimental Section and illustrated in Figure S35, Supporting Information). Furthermore, the TPU hybrid gel can be processed using lithography techniques to fabricate patterned flexible strain sensors, each with different designs to meet various requirements in strain measurement. For example, sensors with longitudinal serpentine lines can measure bidirectional strains, circular designs can measure omnidirectional strains, and strain gauge rosette can determine primary strain and its direction. These different designs enable greater flexibility in the types of strains that can be measured and provide more accurate and precise measurements (Figure S36, Supporting Information).

The electromechanical performance of the patterned soft strain gauge was further quantitated. Figure S37, Supporting Information, exhibited the related resistance change of the soft strain gauge substantially increased with the applied tensile strain. The sensitivity (GF) of the sensor was calculated to be 4.26 at high linearity, which was approximately four times higher than that of the TPU hybrid gel and its materials' intrinsic sensitivity. Additionally, the patterned soft strain gauge was able to operate through thousands of cyclic loading–unloading tests.

**Figure 4:**
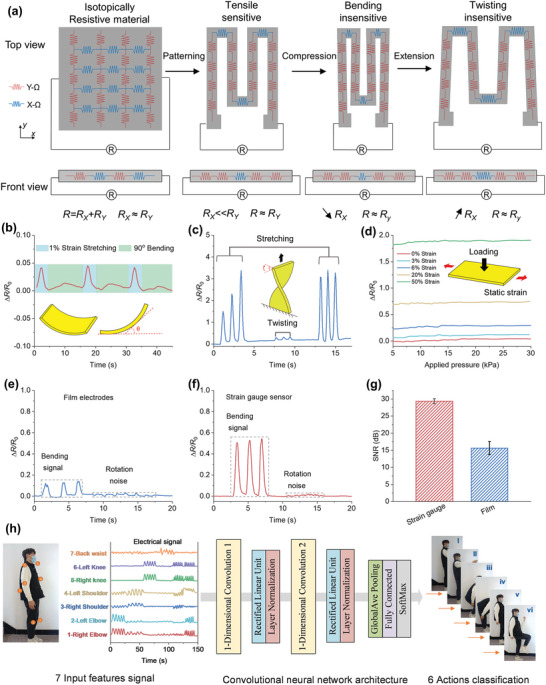
a) Uniaxial soft strain gauge operating mechanism. The influence of off‐axis deformation on sensor performance. Soft gauge sensor detected sequential b) stretching (1% tensile strain) and bending (90°). c) Demonstration of the robustness to off‐axial twisting. The insets illustrate a soft strain gauge under uniaxial stretching and manual twisting. d) Strain gauge detects successively increased pressure for various original strains. The inset illustrates a schematic depiction of the tests. Signal response of e) TPU hybrid gel electrodes and f) soft strain gauge attached on the wrist during repeated bending/rotation movements. g) SNR comparison of TPU hybrid gel electrode and soft strain gauge. h) Signal response of soft strain gauge for full‐body movement monitoring. Multi‐channeled soft strain gauge data were acquired to construct a high‐accuracy neural network model for movement classification.

To further predict the performance of the strain gauge sensor, its mechanism was investigated and a response model was developed. **Figure** **4**a illustrates the electrical sensitivity behavior of the uniaxial strain gauge sensor. The sensor utilizes a soft, isotopically resistive response material that was patterned in the shape of a serpentine meander to enhance its electromechanical robustness. The quasi‐1D structure of the strain gauge endowed it with a nearly ballistic‐resistant response ability. With this structure, the overall electrical resistance was dominated by the uniaxial stretching effect, making it insensitive to the off‐axis deformation.

The unidirectional strain gauge structure had the essential advantage of enabling high linear sensitivity while also combining damage resistance and robustness. It is noteworthy that tests utilizing TPU hybrid gel electrode as the sensing  element have demonstrated extremely intrinsically mechanically robust. Nevertheless, although mechanically compliant, the electromechanical performance of these devices was vulnerable to interference deterioration and potential failure when exposed to adverse operating conditions, such as off‐axis deformations or impact loading. The prepared soft strain gauge structure with a linear sensitive response supported intrinsic hard sensitivity to off‐axis deformation loading. The unidirectional TPU hybrid gel‐based strain gauge was able to detect bending and stretching as illustrated in Figure 4b and Figure S38, Supporting Information, demonstrating that the peak value response in 90° bending being less than 1% of that in stretching. Notably, the 90° bending response single presented negligible peaks compared to 10% tensile strain loading (Figure S39a, Supporting Information). Furthermore, even with extreme bending states of 180°, the strain gauge sensor demonstrated insensitivity to applied loading, with a variation in the electrical signal of less than 6% of the entire operating range (Figure S39b, Supporting Information). The performance of the strain gauge to torsion and tensile extension about the sensor length axis was also evaluated. Figure 4c reveals that off‐axial deformations such as manual twisting did not influence the sensing response, exemplifying the great insensitivity and decoupling deformation capability of the prepared soft strain gauge against torsion disturbance. Furthermore, the soft strain gauge sensors were able to withstand situations that required robustness and damage resistance, such as impact pressure from a hammer strike (Figure S40, Supporting Information). In practical application scenarios, sensing devices might be subjected to external deformation while acquiring uniaxial stretching strains, for instance, in upper‐extremity wearable electrics when the volunteer was lifting weight. Consequently, the capability to simultaneously against these unconcerned deformation influences should be evaluated. The strain gauge sensor in Figure 4d exhibited insensitivity to successively subjected pressures up to 30 kPa at different tensile strain values, with negligible variation in the corresponding electrical signal.

Hence, the soft strain gauge sensor exhibited outstanding sensing performance in acquiring high‐resolution data with high SNR. In contrast, the TPU hybrid gel electrodes in a simple structure, typically fabricated as a rectangle film or bulk, could only record static physiological signals and were unable to acquire valuable proprioception parameters for human movement detection. As illustrated in Figure 4e, when TPU hybrid gel film electrodes were attached to the wrist, the response signals collected from joint bending motion were small and undistinguished from noises (such as joint twisting). However, as released in Figure 4f, by recording the strain‐resistant response of the wrist joint during repeated bending/rotation motions, we demonstrated the high SNR of the strain gauge structure. Similar results were obtained in back bowing/twisting and elbow bending/wagging motion (Figure S41, Supporting Information). The motion signal detected by the strain gauge had an SNR of 29.33 ± 0.75 dB, which was higher than the 15.63 ± 1.88 dB of the TPU hybrid gel film (Figure 4g). Notably, with a unidirectional strain response mechanism to reject the unconcerned movement, the prepared soft strain gauge was appropriated to match the strain changes of various human joints, and the proprioception information can be precisely acquired to classify, track, and reconstruct human movements.^[^ ^43^ ^]^

We also establish seven soft strain gauge sensors on the primary joints of the volunteer subjected (shoulder joint, elbow joint, knee joint, and back) to successfully detected body postures during motion movement. To conveniently transmit the multi‐channel acquired data to the terminal analytical equipment, the seven TPU hybrid gel‐based unidirectional strain gauges were interfaced with data acquisition and Bluetooth integrated devices to fabricate a wireless sensor module (Corresponding equivalent circuit as illustrated in Figure S42, Supporting Information). A large dataset of human motion data was collected using the prepared multi‐channel soft strain gauge (Figures S43 and S44, Supporting Information). The acquired sensor data were preprocessed by normalization and transformation into a standard format that can be efficiency handled by the convolutional neural network (CNN) algorithms. The dataset was divided into a training set and a testing set. The CNN was then trained on the training set and evaluated on the testing set to assess its performance. Once the CNN was validated and its performance was deemed satisfactory, it could be integrated with sensor system applications to classify human motion. The system was capable of acquiring and recognizing seven sensor signals and classifying the data into a variety of six movement states (including left/right elbow lifting, left/right shoulder lifting, squatting, stooping, walking, and running). The convolution neural network architecture achieved a precision of 100% for full‐body movement classification, without incorporating extra vision technologies. The data group exhibited several distinct characteristics that could be used to identify actions and postures. This integrated system incorporating strain gauges shows promise in the monitoring of full‐body motion and tracking of body coordination in sports.

## 3 Conclusions

In this work, we developed a mechanically interlocked gel–elastomer with excellent mechanical and electrical performance. The method employed was the infiltration of a hydrogel precursor into a porous TPU fibers network, which resulted in a distinctive structure that efficiently transfers stress during stretching, leading to high mechanical strength over a board strain range. As a consequence, the TPU hybrid gel exhibited resistance against structural damage formation and crack propagation. Additionally, the remarkable electromechanical performance of the TPU hybrid gel electrode in the fast limit detection and full range dynamic vibration response, enabling the electrode to monitor and evaluate physiological signals related to human activity. Meanwhile, the manufacturability of TPU hybrid gel electrodes can expand their ability to sense physiological signals. The laser‐cut fabricated TPU hybrid gel‐based soft strain gauge showed high SNR and electromechanical robustness, allowing for accurate human movement recognition and classification with the assistance of machine learning techniques. This approach, which combines the distinct yet complementary advantages of dissimilar materials, can be applied widely to wearable electronics. The novel gel–elastomer hybrid material and devices developed in this study offer new possibilities for the Internet of Things and other intelligent applications.

## 4 Experimental Section

### Materials

TPU was purchased from Shanghai Aladdin Biochemical Technology Co., Ltd. PVA and acrylic acid (98%) were supplied by Tansoole.

### Fabrication of Electrospinning TPU Substrate

The electrospinning solution was prepared by incorporating TPU pellets into a mixed solvent of tetrahydrofuran and dimethyl formamide in a mass ratio of 4:1, and then stirring for 10 h to obtain a homogeneous transparent precursor. Electrospinning was applied at a feeding rate with the volumetric flow at 2.0 mL h^−1^. A rotating drum was adopted as the collector at a speed of 50 r min^−1^, and the distance between the spinneret tip to collect was 14 cm.

### Synthesis of PVA/P(AAm‐co‐AA)‐CaCl_2_ Ionic Gel Matrix

The ionic gel matrix was synthesized by mixing 25 mL of a precursor solution of 1 wt% PVA, 4 wt% acrylic acid, and 16 wt% acrylamide monomers, 0.016 wt% *N*,*N*′‐methylene bisacrylamide and 0.8 wt% the photoinitiator. Then various weight fractions of CaCl_2_ (10, 20, and 30 wt%) were incorporated into a fully dissolved solution to obtain the gel components. The gel precursor was degassed by pouring it into a vacuum oven and slowly evaporated in an opening ambient to form the ionic gel matrix for infiltrating the TPU substrate.

### Preparing of Mechanically Interlocked TPU Hybrid Gel

First, the TPU substrate was treated with oxygen plasma to make it hydrophilic. This step was aimed at modifying the surface properties of the TPU substrate to improve its compatibility with the ionic gel matrix. Then, the ionic gel matrix was meticulously cast onto the oxygen plasma‐treated TPU. The gel precursor solution was filled into the porous TPU substrate by capillary action, and by utilizing vacuum degassing to further remove the air in porous TPU and made full contact between the ionic gel and TPU. Finally, the TPU hybrid gel was obtained by polymerizing the ionic gel matrix using a UV light for 1 h.

### Manufacture of TPU Hybrid Gel Based Soft Strain Gauge

Electrospinning TPU substrate was attached to a steel plate and patterned with laser cutting (523 nm), acting as the template for polymerization of PVA/P(AAm‐co‐AA)‐CaCl_2_ ionic gel matrix. Then, the patterned TPU was treated with oxygen plasma and slowly filled with a liquid mixture of ionic gel through capillary action. The resulting TPU structure was covered with the conductive gel and subjected to UV light, ultimately producing a soft strain gauge.

### Electrophysiological Signals Recordings

The TPU hybrid gel electrodes were attached to the volunteer in pairs, and ECG signals were obtained using an RS‐EMG4 (Iwork) device. Three TPU hybrid gel unipolar electrodes were attached to the hand to obtain EMG signals for hand gesture recognition.

SNR was calculated from the equation

(1) $$\mathrm{SNR}\left(\mathrm{dB}\right)=20\log10\left(\frac{A_{\mathrm{signal}}}{A_{\mathrm{noise}}}\right)$$

where *A*_signal_ and *A*_noise_ denote the peaks of the response signal (concerned movement) and background (unconcerned movement), respectively.

Soft strain gauges based on TPU hybrid gel were used to monitor the strain changes of a series of volunteer joints. The movements of the body were recorded using a multichannel electrical signal acquisition (DAQ, TruEbox‐01RC). The raw data collected by the sensors were analyzed to extract features that could be utilized to train a machine‐learning algorithm capable of classifying different types of movements and detecting changes in movement patterns over time. The accuracy and effectiveness of the algorithm were evaluated once it had been trained. To conduct related human motion detection experiments on the human body, rules or permissions from the relevant national or local authorities are not in place in the country where the experiments were performed. The volunteers took part in this proof‐of‐concept illustration with a full understanding of the process and any risks involved. Informed written consent was obtained from the volunteers.

## Conflict of Interest

The authors declare no conflict of interest.

## Supporting information

Supporting Information

Supplemental Video 1

Supplemental Video 2

Supplemental Video 3

Supplemental Video 4

Supplemental Video 5

Supplemental Video 6

Supplemental Video 7

## Data Availability

The data that support the findings of this study are available from the corresponding author upon reasonable request.
