# Supplementary material for: Tough and Robust Mechanically Interlocked Gel–Elastomer Hybrid Electrode for Soft Strain Gauge
Source: Adv Sci (Weinh). 2023 May 3;10(21):2301116. doi: 10.1002/advs.202301116 (PMC10375198; doi:10.1002/advs.202301116)
Supplement: Supplementary file 1 — Supporting Information [file ADVS-10-2301116-s004.pdf]

ADVANCED SCIENCE
Open Access

Supporting Information

for *Adv. Sci.*, DOI 10.1002/advs.202301116

Tough and Robust Mechanically Interlocked Gel–Elastomer Hybrid Electrode for Soft Strain Gauge

Jianren Huang, Anbang Chen, Songjiu Han, Qirui Wu, Jundong Zhu, Jiayu Zhang, Yujia Chen, Jiantao Liu and Lunhui Guan**

Supporting information

Tough and Robust Mechanically Interlocked Gel–Elastomer Hybrid Electrode for Soft Strain Gauge

Jianren Huang, Anbang Chen, Songjiu Han, Qirui Wu, Jundong Zhu, Jiayu Zhang, Yujia Chen, Jiantao Liu, Lunhui Guan**

J.R. Huang, A.B. Chen, S.J. Han, Q.R. Wu, J.D. Zhang, J.Y. Zhang, Y.J. Chen and Prof. L.H. Guan

CAS Key Laboratory of Design and Assembly of Functional Nanostructures, Fujian Key Laboratory of Nanomaterials, Fujian Institute of Research on the Structure of Matter, Chinese Academy of Sciences, Fuzhou 350108, China.
E-mail: guanlh@fjirsm.ac.cn

Dr. J.T. Liu
Xiamen Port Holding Group co. Ltd, Xiamen, Fujian, China

E-mail: liujiantao@gmail.com

Supplementary Note 1. Characterizations and Measurements

Material Characterizations: In the characterization of the ionic gel and TPU hybrid gel, the Field-Emission Scanning Electron Microscopy (Verios G4, America) instrument was utilized, and it was operated at an acceleration voltage of 5 kV. The FT-IR spectrometer (Vertex 70, Bruker) used in this study was operated in the wavenumber range of 4000–600 cm^{-1}, which covers a broad range of the infrared region and enables the detection of a wide range of functional groups. For preparing FT-IR samples, the TPU hybrids gel was first cut into small pieces, dried in an oven, and then ground into a fine powder. Subsequently, the hybrid gel powder was mixed with potassium bromide (KBr) and compressed under high pressure to form a thin pellet. Finally, the blank group (without specimen) was calibrated before testing the specimen. The pellet was placed in the sample holder of the FT-IR spectrometer and analyze in accordance with the instrument manufacturer's instructions. Raman spectra were conducted on a Dilor LABRAM-1B multi-channel confocal micro-spectrometer with a 532 nm laser excitation. Thermogravimetric analysis (TGA) was performed using a TG-DTA instrument (STA449F5) between 25 to 800 °C at a heating speed of 10 $^{o}C\ min^{-1}$ under an argon atmosphere. The hydrophobicity of the membranes was measured with a video water contact angle system (SPCA, HARKE). The ionic conductivity of the gel was investigated by electrochemical impedance spectroscopy, and the ionic conductivity σ was calculated using the following equation:

$$\sigma = \frac{L}{RA} \quad (1)$$

In the formula, L is the thickness of the electrode film, R is the bulk resistance obtained from the EIS plot, and A is the area of an electrode.

Tensile Testing: The TPU hybrid gel specimens were cut into dumbbell-shaped with a gauge width of 3 mm for uniaxial stretching tests. The thickness of each sample was quantitated with a caliper and was approximately 0.15 mm. The stress-strain curves were acquired using a universal material test machine (AG-X plus, SHIMADZU, Japan) equipped with 100 N loading cells. The mechanical property was evaluated at room temperature.

Pure shear test: The test configuration proposed by Greensmith has been recently utilized t to determine the fracture energy Γ [1]. Dumbbell-shaped samples with and without notches were used for testing, with the unnotched sample used to measure the stress-strain curve. The energy release

rate for single edge notch was approximately calculated using a compliance method and the following equation:

$$\Gamma = \frac{6}{\sqrt{\lambda_b}} W(\varepsilon_b) c \quad (2)$$

Where λ_b was the fracture deformation ratio of notched TPU hybrids gel ($\lambda_b = \varepsilon_b + 1$), $W(\varepsilon_b)$ was the strain energy density of an unnotched sample applied to a uniaxial strain ε_b, c is the length of the notch.

Fatigue test: Similar single-notch tensile tests were carried out to verify the fatigue resistance of the specimens. The experimental equipment was illustrated in Figure S13. Cyclic stretching tests were performed by using notched samples with various precut crack lengths (*c*), which are smaller than the width (*D*) of the sample. All tensile cycles were continuously conducted at a speed rate of 20 mm/s under various strains without any relaxation time.

The energy release rate (*G*) was calculated by the following equation:

$$G = 2kcW \quad (3)$$

Where *k* was a related function of strain variation empirically determined by $k = 3/\sqrt{\varepsilon + 1}$, *c* was the crack propagation length, and *W* was the strain energy density of an unnotched sample of the same dimensions stretched to the same strain ε.

Vibration test system: To establish a measurement system capable of generating vibrations with controllable frequency and measuring the amplitude, the appropriate equipment for the measurement system, such as a signal amplifier (UTEKL YE1311), vibration exciter a (UTEKL JZ-1), accelerometer (Bruel-Kjaer DeltaTron 4520-001), and a data acquisition system (UTEKL 3404FRS-DY) to collect and store the data, were configured. And then, perform test runs and collect data, adjusting the parameters as needed to obtain the desired frequency range and amplitude levels. The collected data were analyzed to determine the frequency of the vibrations under various loading conditions.

Electromechanical Response Measurements: the electrical resistance under the applied mechanical strain of the electrodes/sensor were acquired continuously by a digital SourceMeter (Keithley 2450, Tektronix Co.).

Machine learning: Convolutional layers were employed to recognize significant patterns and

features in the data that were pertinent to the classification task. These extracted features were fed into fully connected layers that perform the actual classification task. The output of the fully connected layers was a probability distribution over the different classes of human motion. The CNN was trained using a labeled dataset of human motion data.

Supplementary figures and tables

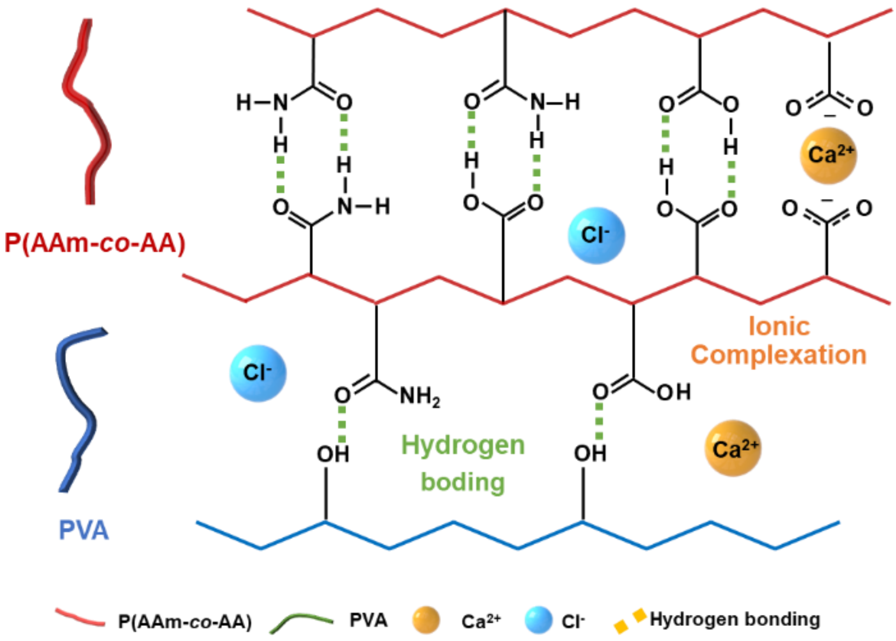

Figure S1. Multiple molecular interactions in prepared ionic gel.

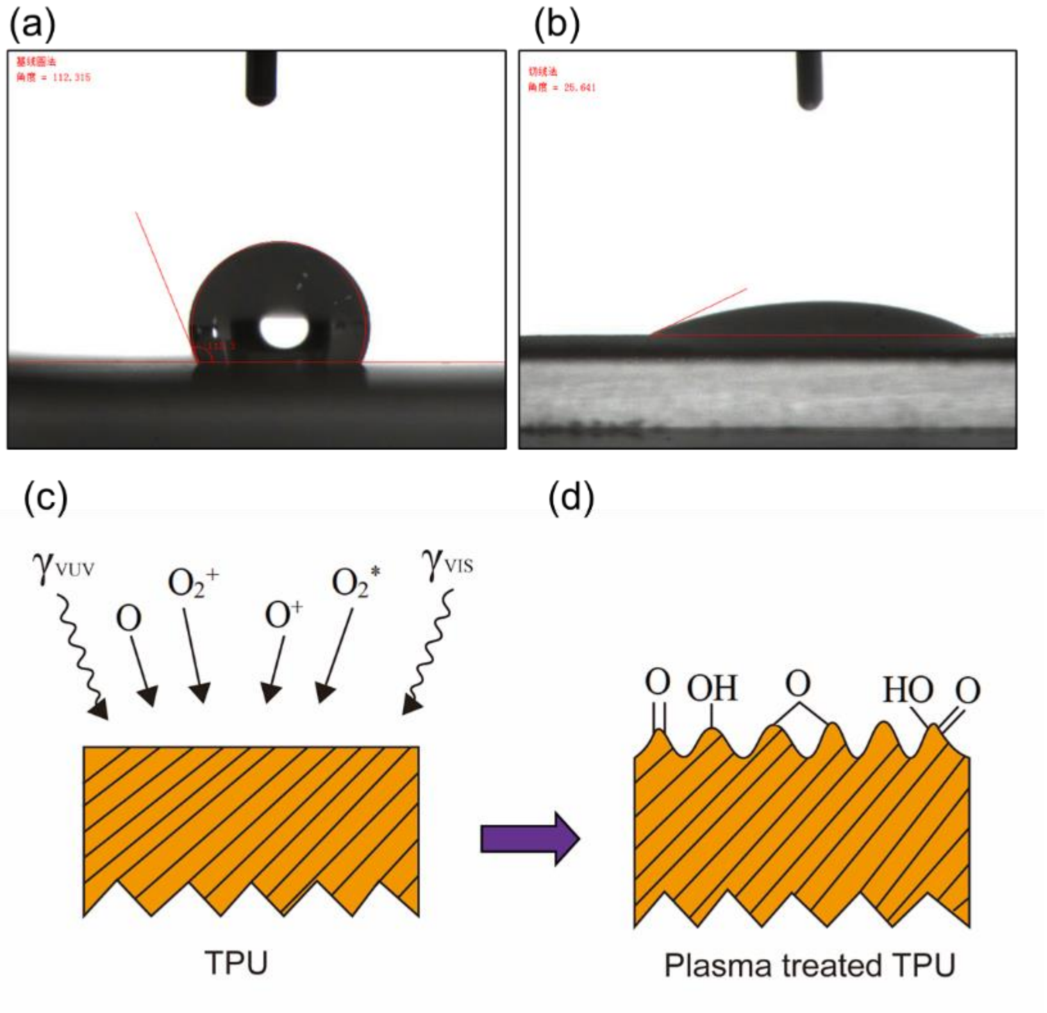

Figure S2. The photographs show the water contact angle of (a) TPU substrate and (b) oxygen plasma treated TPU film (c) initial smooth TPU surface. (b) TPU after exposed to oxygen plasma assumes rich morphology and becomes functionalized with polar surface groups.

To quantify the hydrophilic of the TPU substrate, water contact angle measurements are carried out. The TPU substrate is hydrophobic with an initial contact angle of 112.3° while with the plasma treatment by oxygen, the TPU substrate turns to be highly hydrophilic (25.6°) due to attaching an abundant of functional groups and the droplet could be completely absorbed within 1 s, as illustrated in the Figure S2. The infiltration of the pre-polymerization ionic gel could be greatly enhanced by the hydrophilic characteristic of the TPU, which is critical to the improved adhesion strength between the ionic gel and TPU substrate.

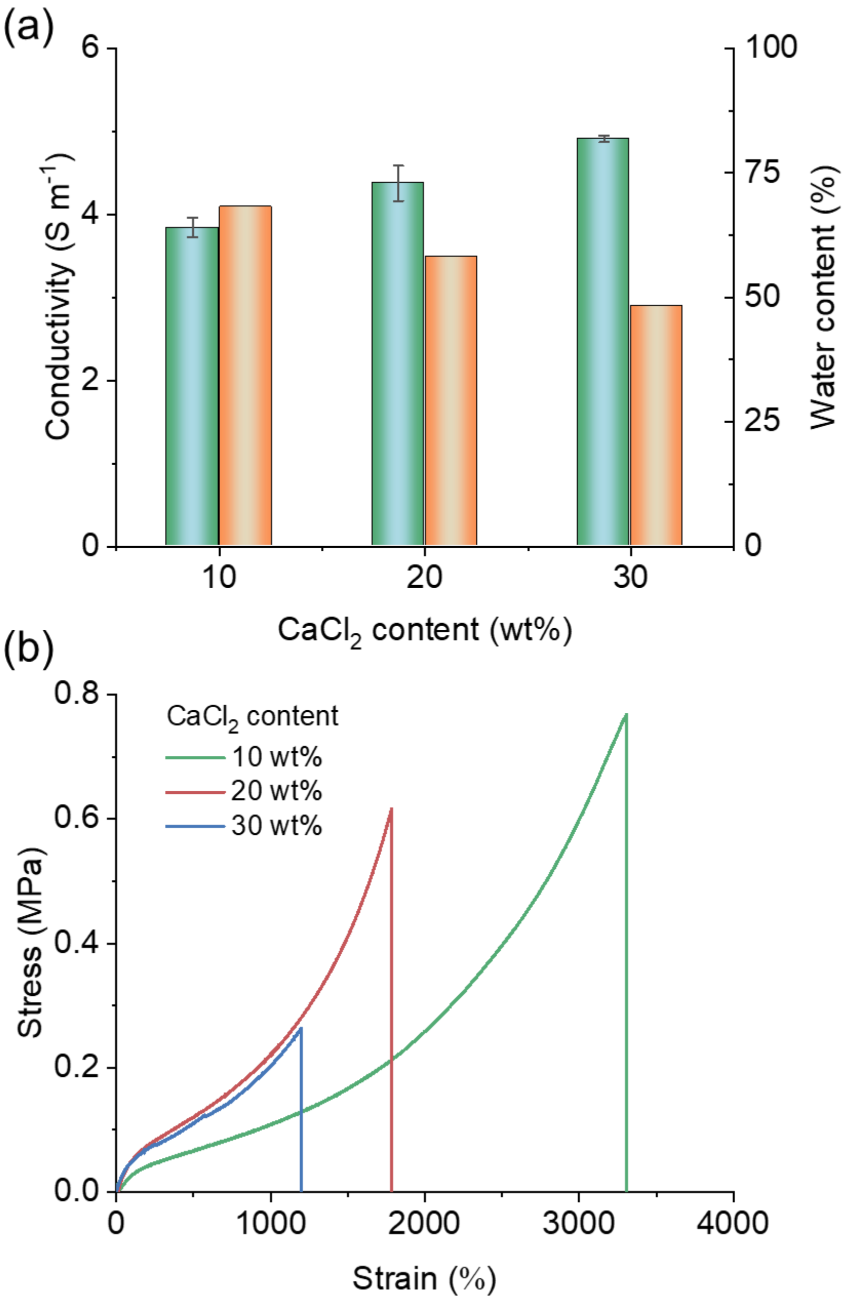

Figure S3. Water contents, ionic conductivities, and stress-strain curves of PVA-P(AAm-co-AA)/$CaCl_2$ ionic gel with different $CaCl_2$ contents. The $CaCl_2$ content is referred to as the weight proportion in the dry samples.

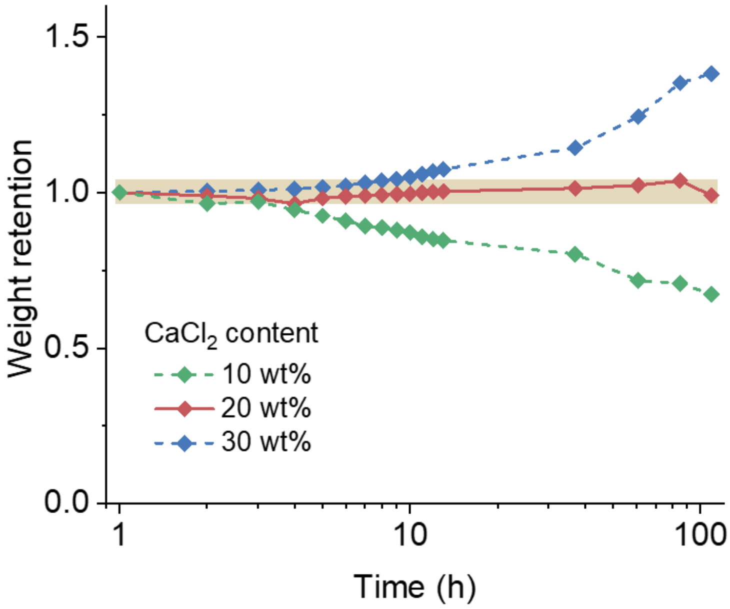

Figure S4. Water retention of the ionic gel with various $CaCl_2$ content at different time intervals.

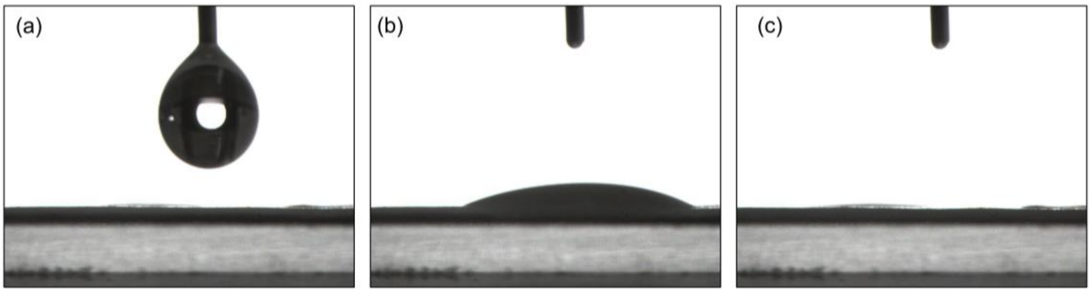

Figure S5. The photographs show the ionic gel infiltration process.

The infiltration process was recorded of the hydrogel precursor by a CCD camera. Due to improved the hydrophilicity of the TPU substrate, allowing the hydrogel precursor rapidly infiltrate to the whole substrate.

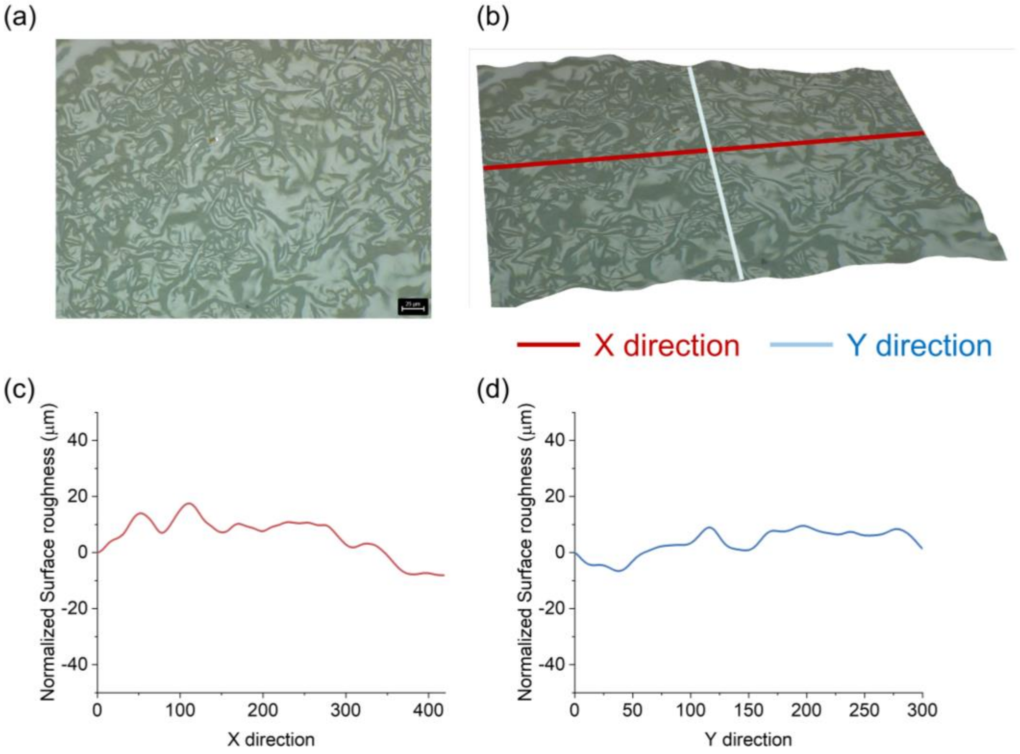

Figure S6. (a) 2D and (b)3D surface microscopic image of TPU hybrid gel. (c) Surface flatness of material in X direction (d) Surface flatness of material in Y direction

The surface morphology was obtained by the ultra depth of field video microscope (Leica DVM6) to investigate the uniformity and consistency. As illustrated in Figure S6a and b, the ions gel was uniformly distributed in the TPU fiber network, and no obvious bubbles and cavities were observed. According to the path in the in the 3D microscopic image to calculate the surface flatness of the TPU hybrid gel in Figure R5c and 5d. Regardless of any direction, the surface of the hybrid gel was relatively flat and without distinct fluctuations. Therefore, we believe that the good hydrophilicity of the plasma treated TPU fibers ensures the uniformity and consistency of infiltration.

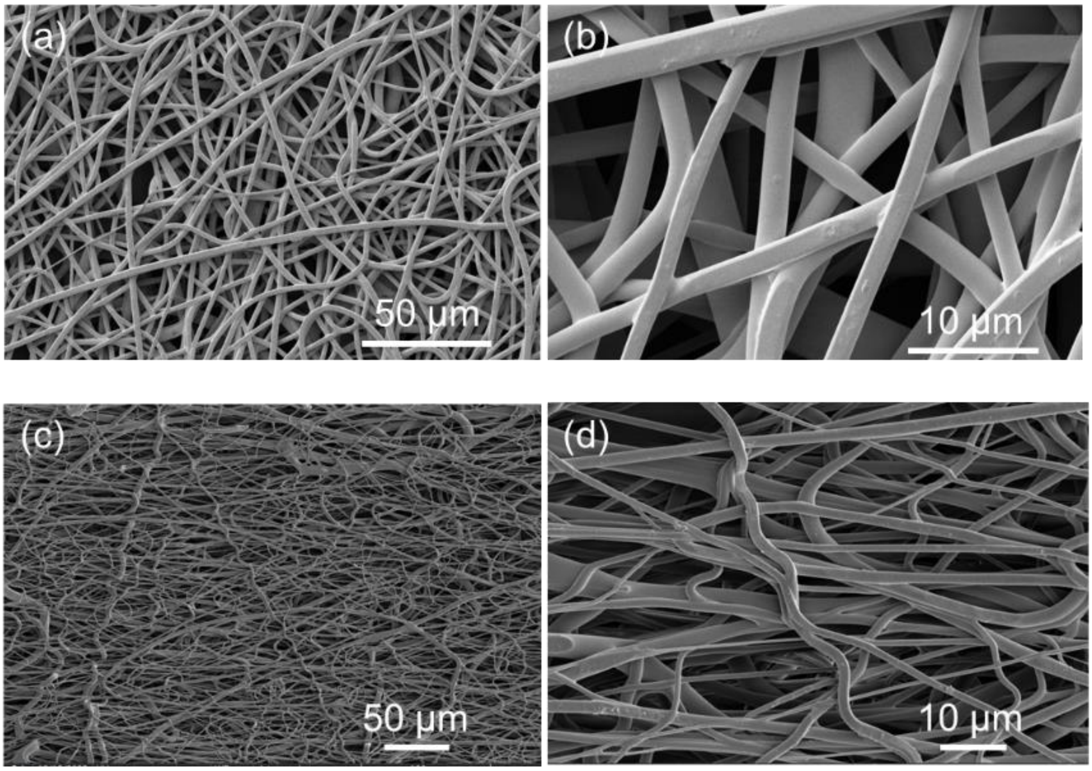

Figure S7. (a) and (b) SEM image of electrospinning TPU thin film. (c) and (d) SEM image of TPU fiber network under a strain of 50% (in horizontal direction).

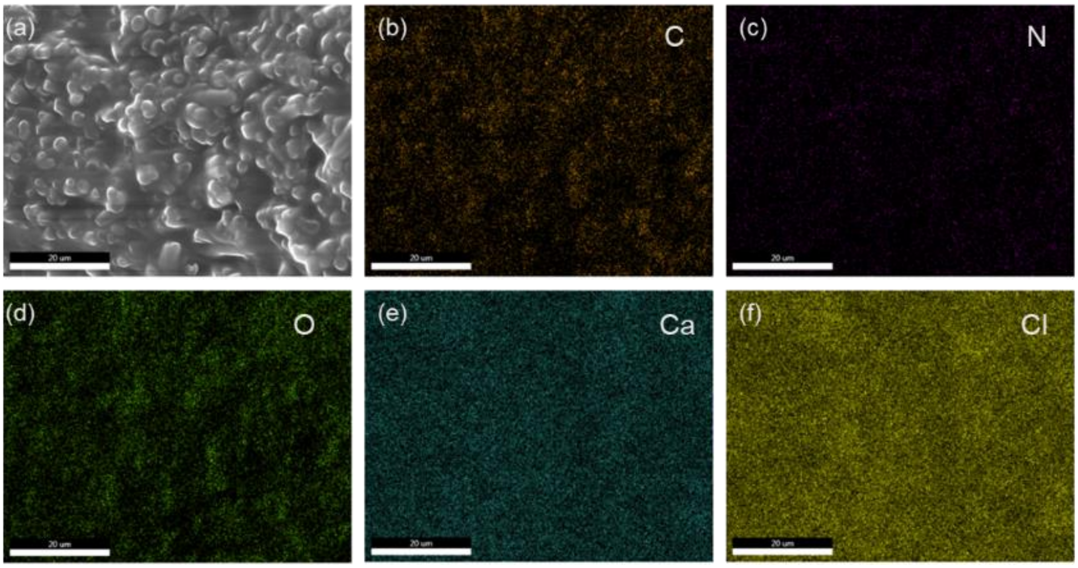

Figure S8 (a)The cross-sectional SEM image of TPU hybrid gel. (b)-(f) corresponding EDS mapping images of TPU hybrid gel

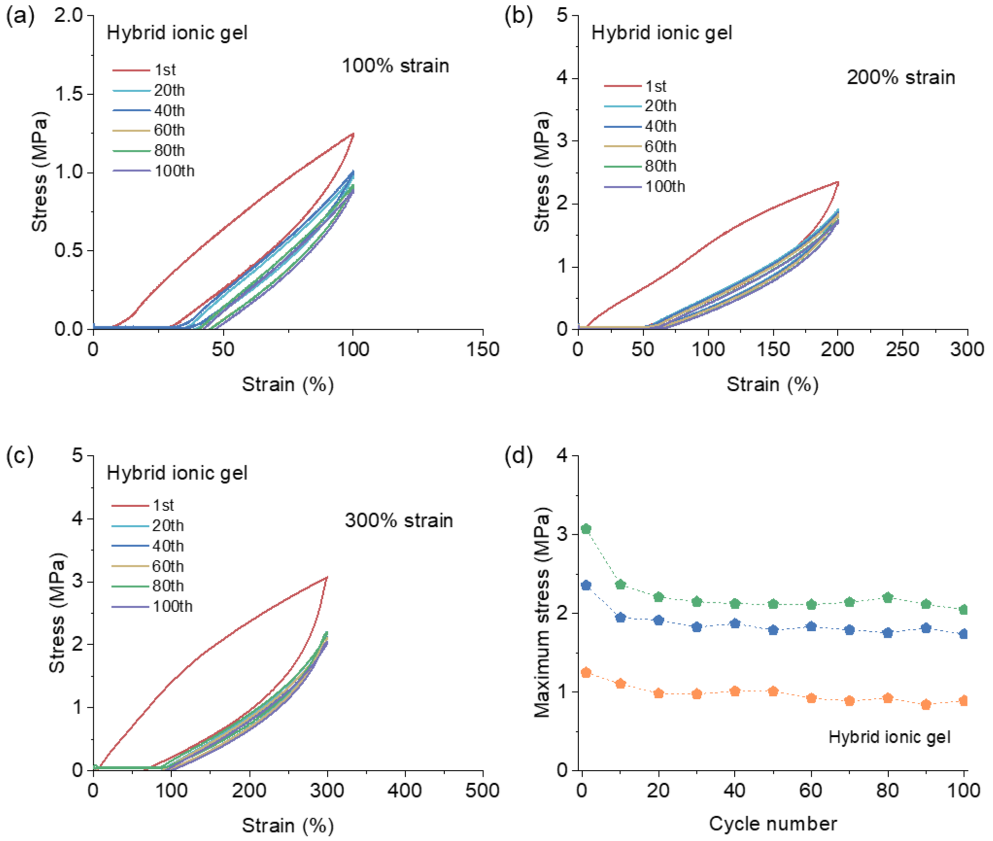

Figure S9. The successive cyclic uniaxial loading-unloading tensile curves of unnotched TPU hybrid gel at different elongation ratios, (a) 100%, (b) 200%, and (c)300%. (d) The Max stress during the cyclic stretching test.

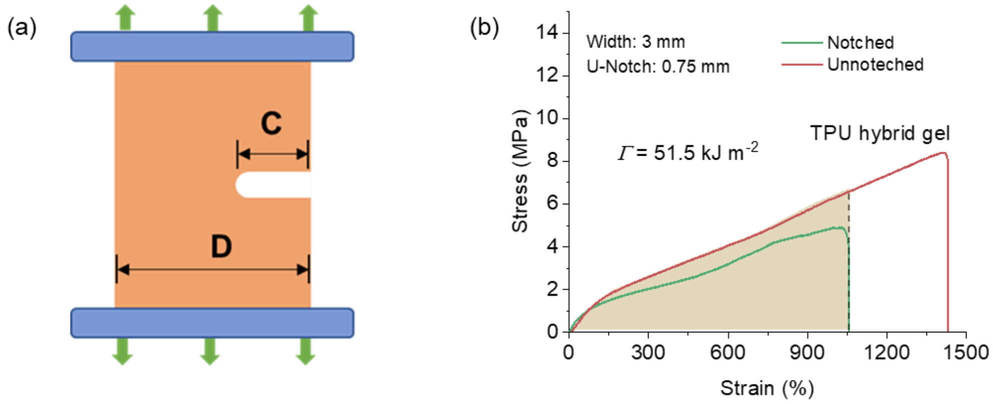

Figure S10. (a) Illustration of a single-notched sample. (b) Stress-strain curves of unnotched and notched TPU hybrid gel. The width of the notched sample is 3 mm, and the length of the circular-type (U-Notch) pre-cut notch on the sample is 0.75 mm.

According to the single-edge notch tension test, the calculated fracture energy of TPU hybrid

gel is 51.5 kJ m^{-2}.

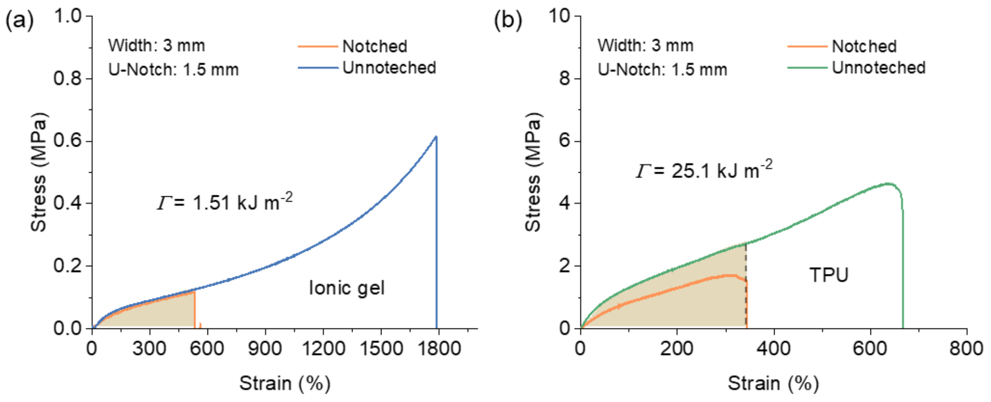

Figure S11. Stress-strain curves of unnotched and notched (a)ionic gel and (b) TPU subtract. The width of the notched sample is 3 mm, and the length of the circular-type (U-Notch) pre-cut notch on the sample is 1.5 mm.

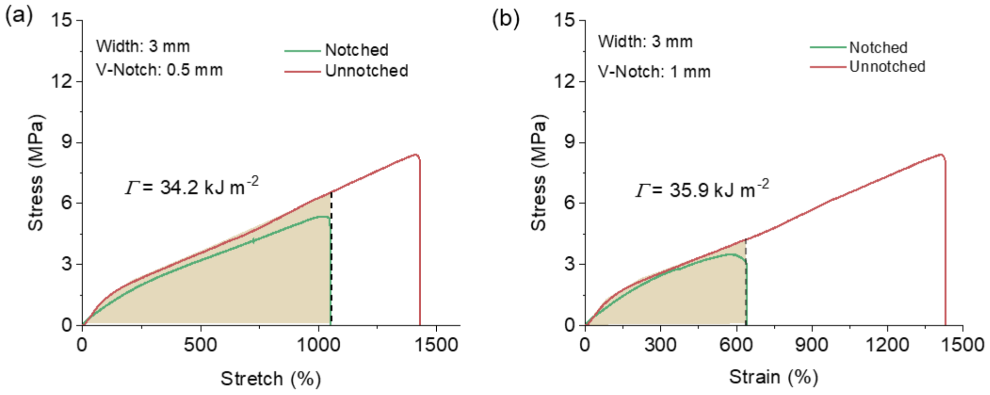

Figure S12. Stress-strain curves of unnotched and notched TPU hybrid gel with different notched sizes. The width of the notched sample is 3 mm, and the length of the linear-type (V-Notch) pre-cut notch on the sample is (b)0.5 mm and (c)1 mm

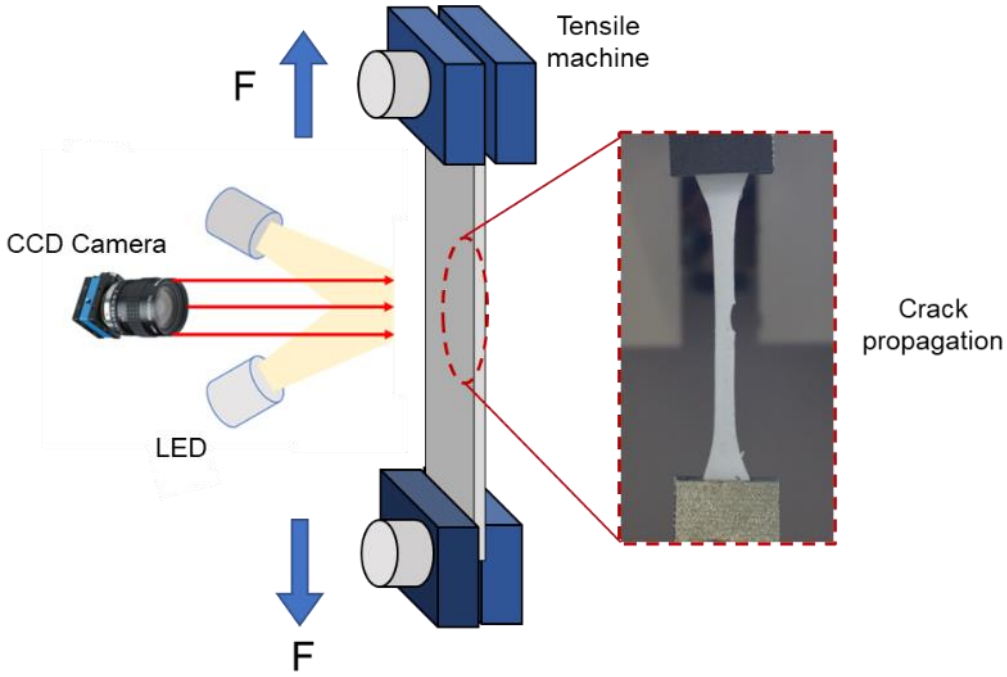

Figure S13. Experimental method and equipment for fatigue and fracture test

We recorded the force-displacement curve of each unnotched sample over many cycles, and plotted the nominal stress as a function of the cyclic loading number. The experimental equipment was illustrated in Figure S13. All experiments were employed by using the same frequency of 1 Hz. The marked ruler line was attached to the surface of the test machine. As the tensile tester pulled the sample cyclically, the crack propagated cycle by cycle and the extension of the crack was recorded by a digital camera (SNOY, a6000) and industrial camera (ON Semiconductor PYTHON5000).

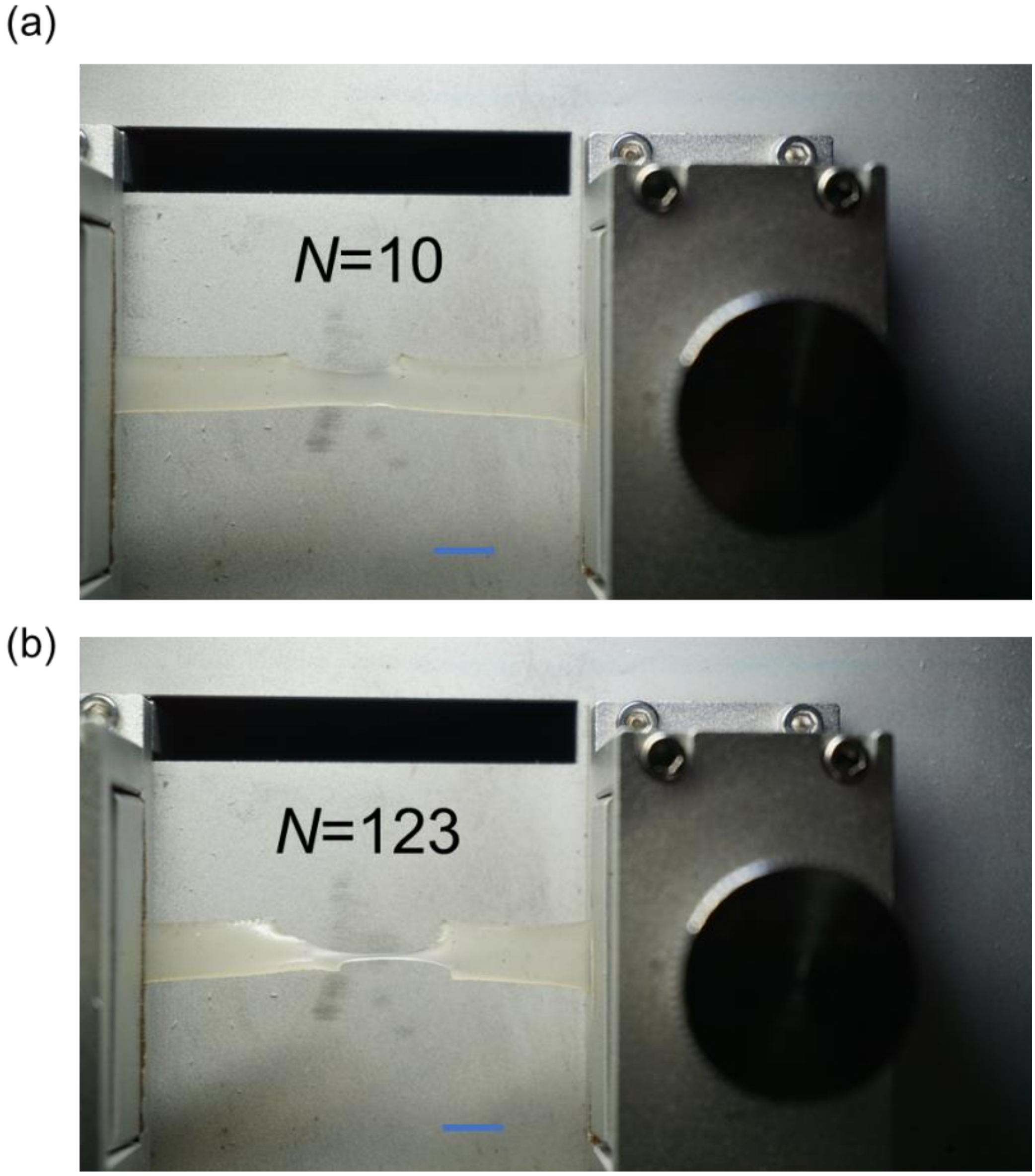

Figure S14. Snapshots at 10 and 123 cycle at a crack in ionic gel subject to cyclic load of stretch of amplitude ε=200%.

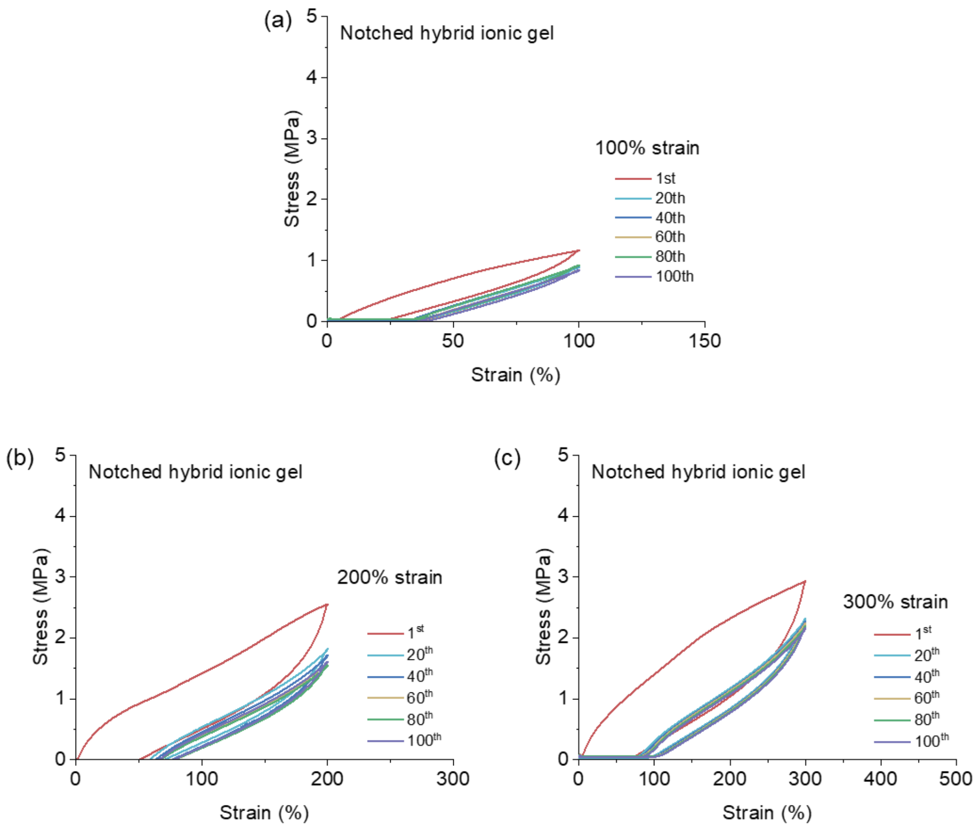

Figure S15. Cyclic uniaxial loading-unloading tensile curves of notched TPU hybrid gel at different elongation ratios, (a) 100%, (b) 200% and (c)300%.

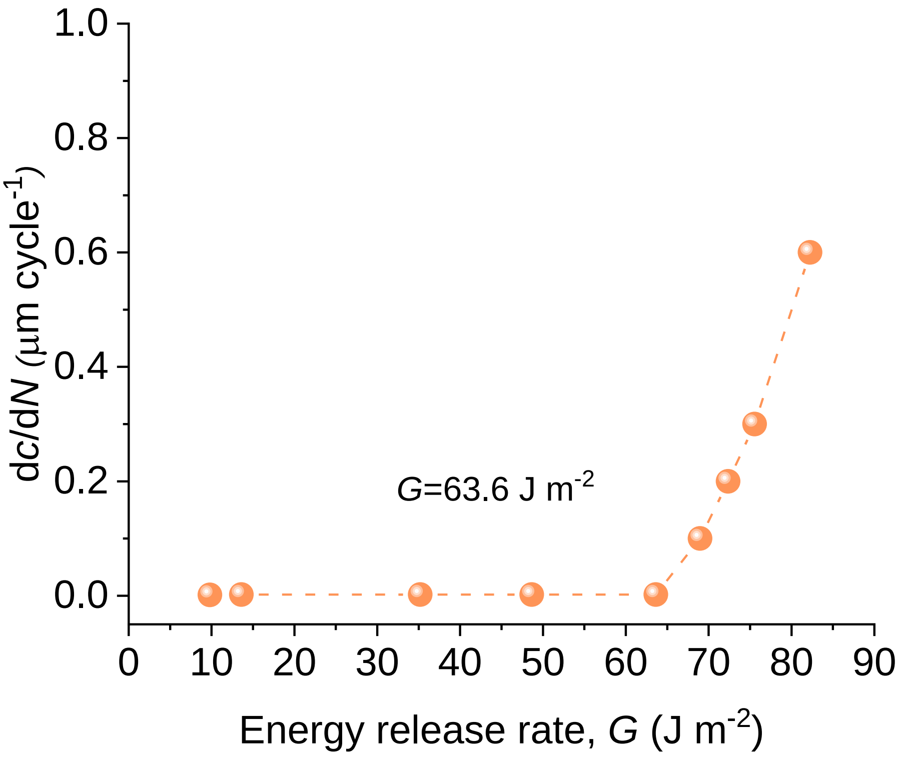

Figure S16. Crack extension per cycle *dc/dN* versus applied energy release rate *G* for ionic gel

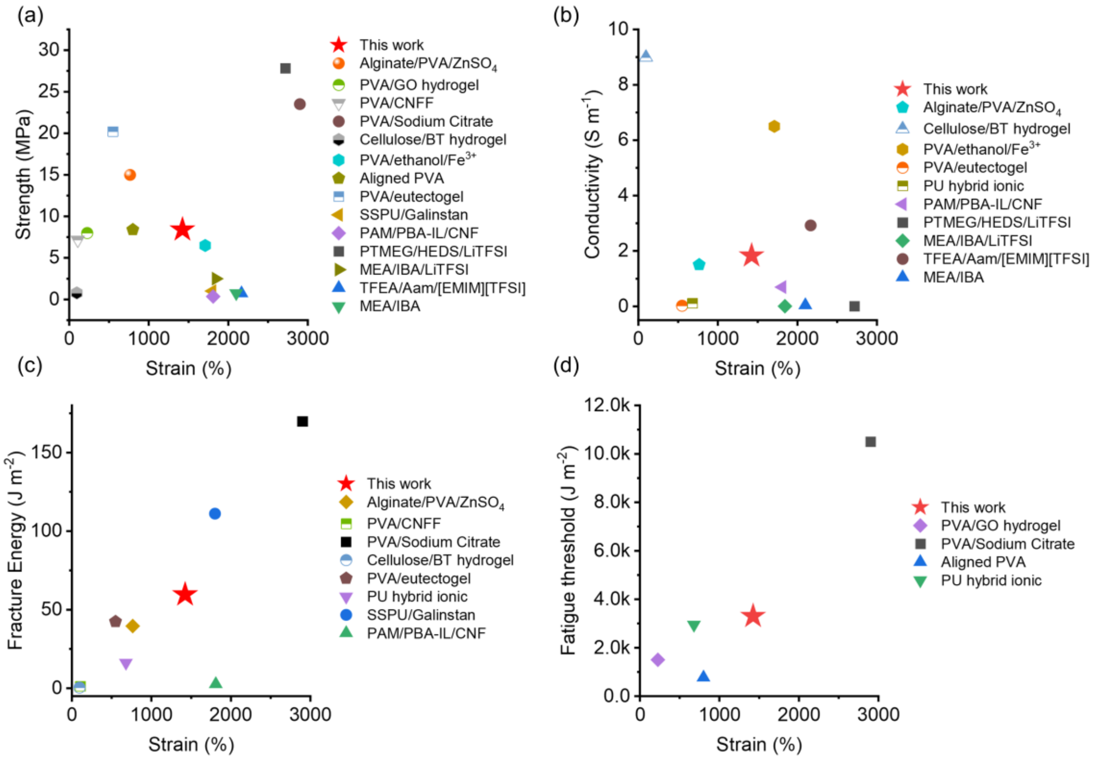

Figure S17. Ashby diagrams of (a) ultimate tensile strength versus ultimate tensile strain, (b) conductivity versus ultimate tensile strain, (c) fracture energy versus ultimate tensile strain and (d) fatigue threshold versus ultimate tensile strain of TPU hybrid gel, other reported tough hydrogels and other elastomer. The data used are summarized in Supporting Information of Table S2.

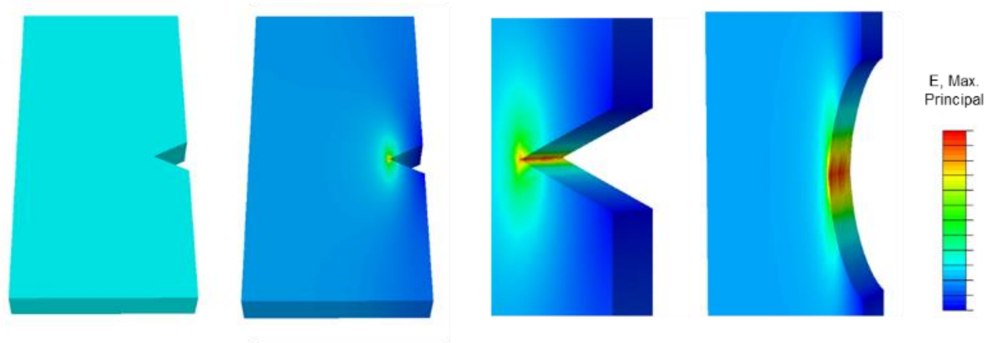

Figure S18. Scale bar from red to blue indicates that the stress concentration from high to low.

Figure S18 illustrated the FEA models of the ionic gel. We assumed different components were isotropic and uniform in integrated overall structure. The Young's modulus and Poisson's ratio of TPU fiber were respectively 614.57 MPa and 0.35, those of the ionic gel mixture are

respectively 10 MPa and 0.4. The structures were discretized using hexahedral elements (C3D8). The binding constraint was applied in different layers with tensile strain loading. The crack propagation process was simulated by the extended finite element method with sweep mesh refinement. As a result, the ionic gel with crack induces severe stress concentration on the crack tips, while the structure with an TPU enhanced induces a slightly stress concentration on notched region.

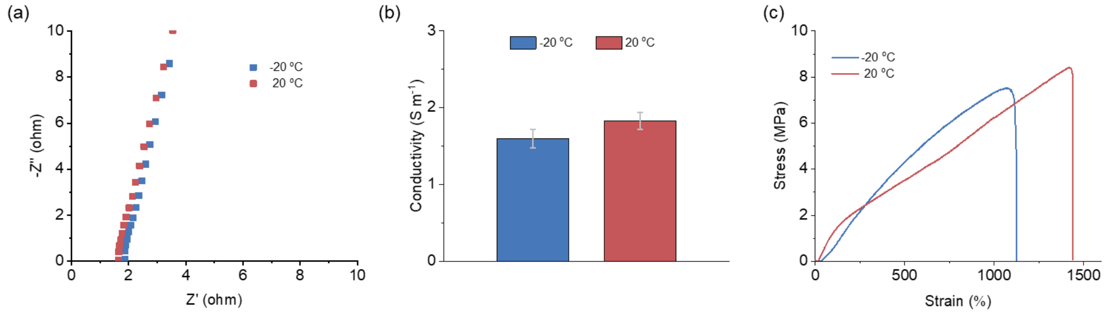

Figure S19. (a) Nyquist plots of TPU hybrid gel at different test temperatures. (b) the conductivity of TPU hybrid gel at different test temperatures. (c)Tensile stress-strain curves of the TPU hybrid gel at 20 °C and -20 °C.

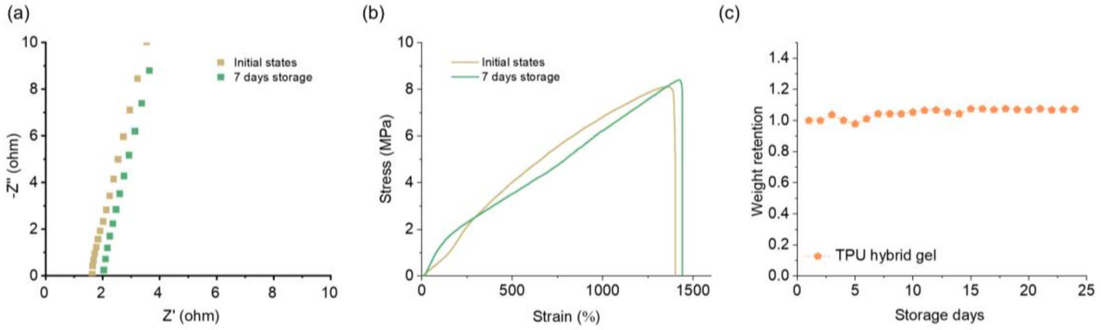

Figure S20. (a) Nyquist plots of TPU hybrid gel with different storage time. (c)Tensile stress-strain curves of the TPU hybrid gel with different storage time. (c)The dehydration process of freshly as-prepared TPU hybrid gel storage at open air atmosphere.

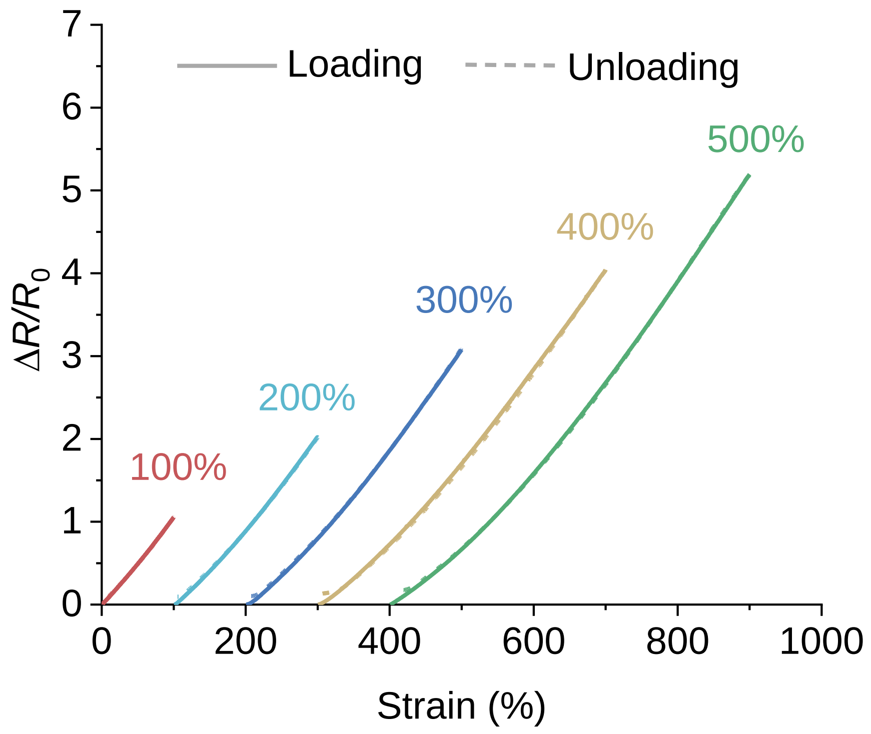

Figure S21. Loading and unloading relative resistance responses of the electrode with various strain range (from 100 to 500%), exhibiting low hysteresis and high linearity.

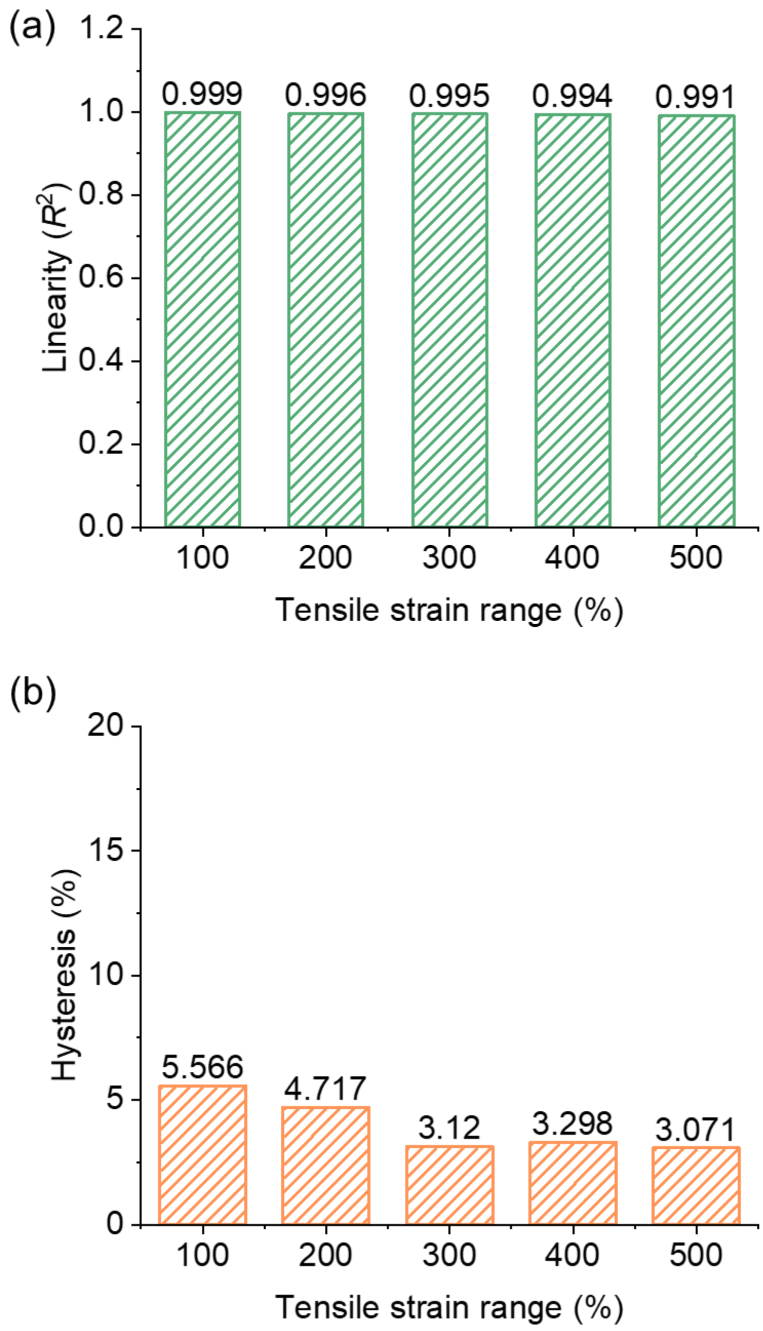

Figure S22. The response (a)hysteresis and (b)linearity of TPU hybrid gel membrane in cyclic Loading and unloading electromechanical tests from 100% to 500 strain%

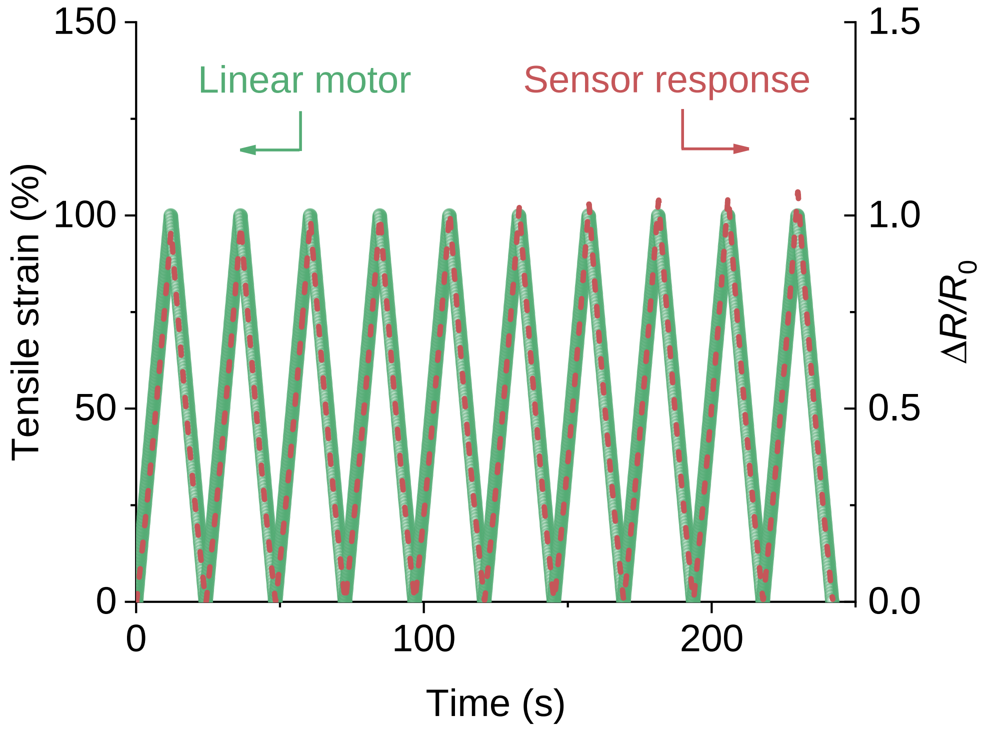

Figure S23. Electrical hysteresis responses curves of the variation in resistance and strain.

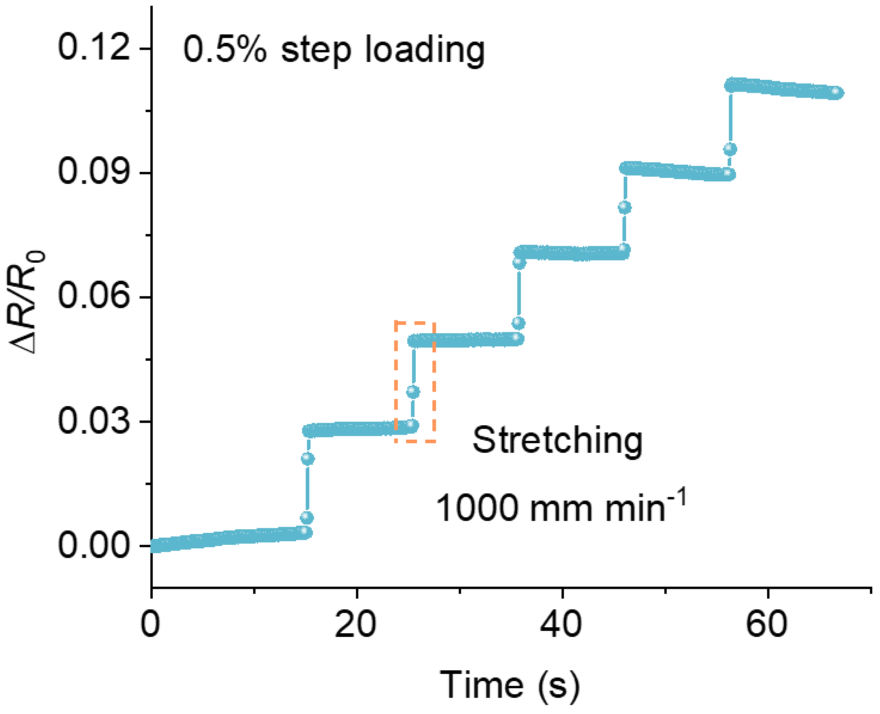

Figure S24. Resistance response of the electrode under a series of step-up strains of 0.5%.

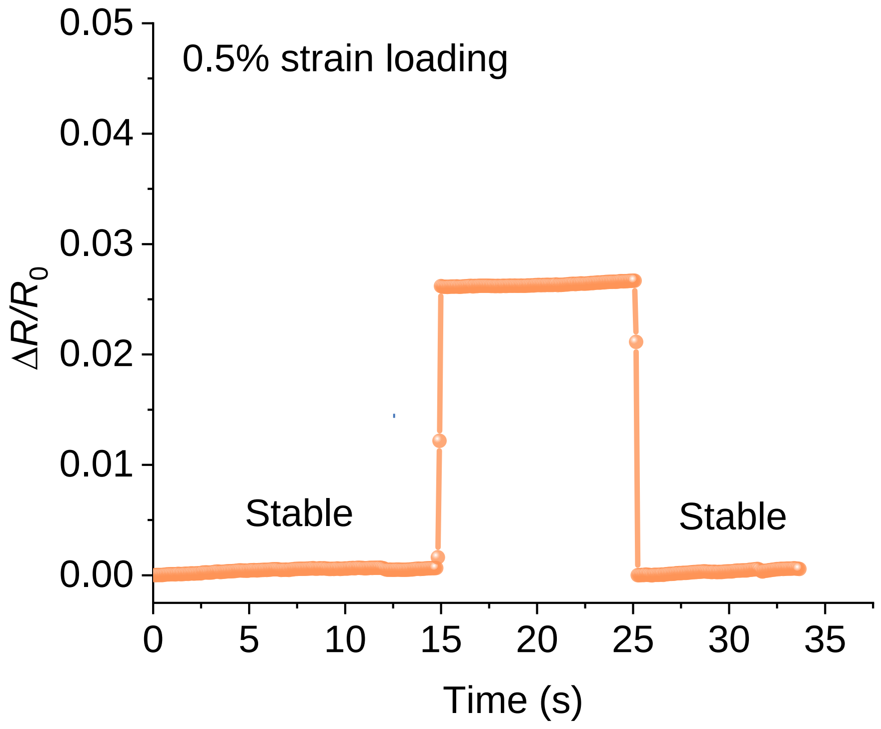

Figure S25. Response stability test of the TPU hybrid gel membrane at 0.5% tensile strain

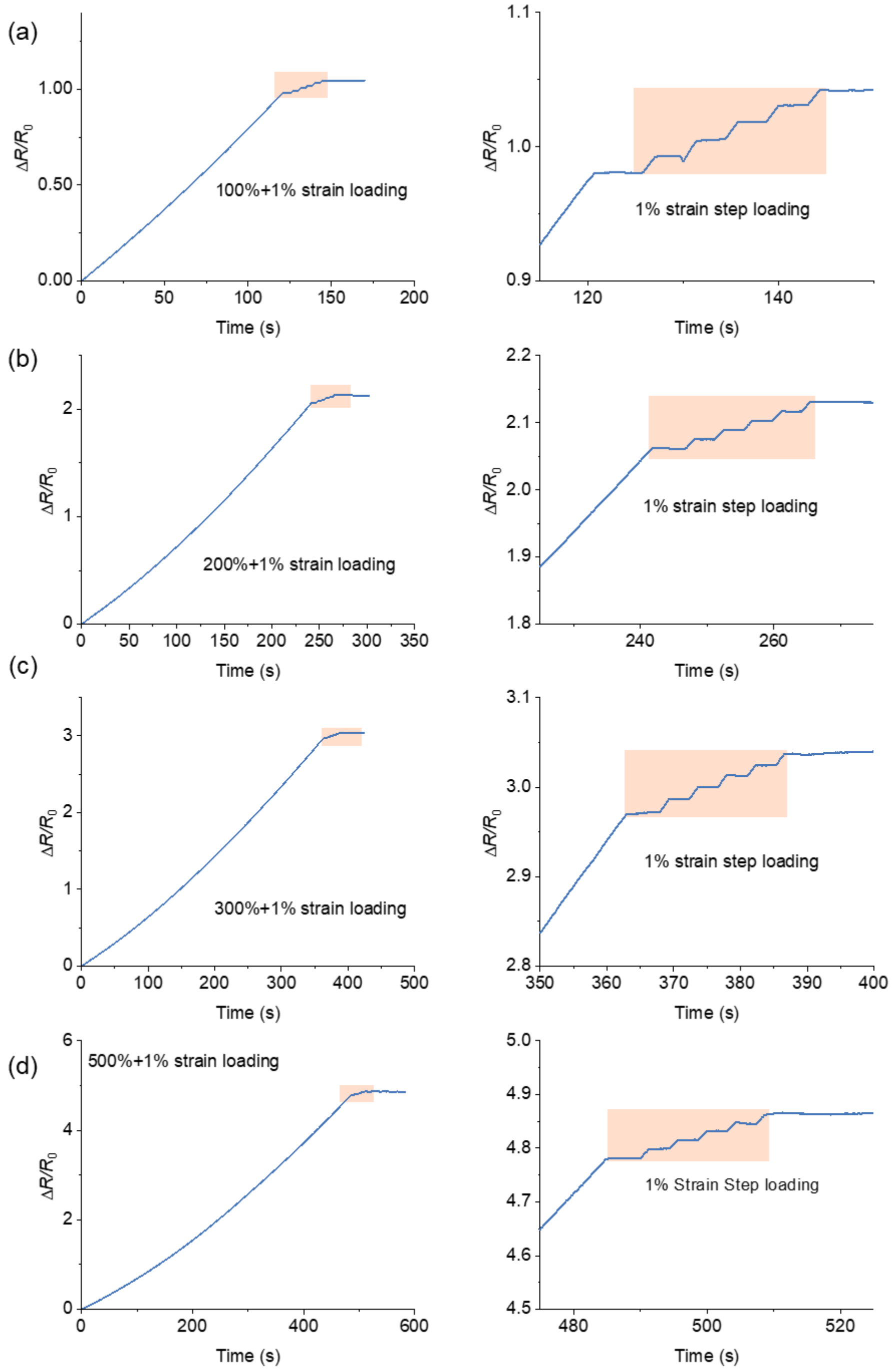

Figure S26. Detection of sequential single-step ultralow strains (0.05%) under a large base strain. a)100%, b)200%, c)300%, d)500%

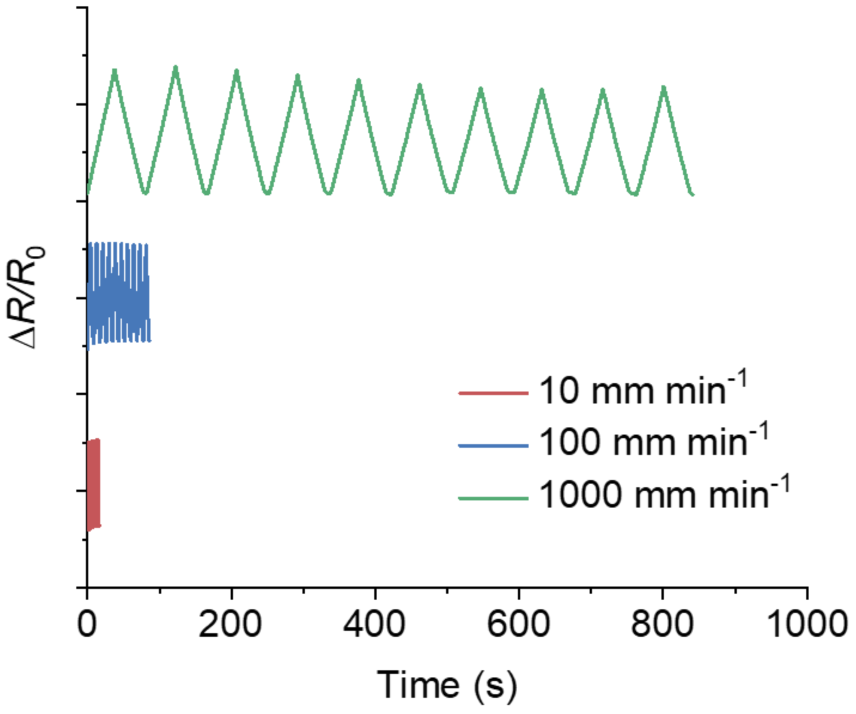

Figure S27 Electromechanical response of TPU hybrid gel membrane under cyclic loading-unloading with a strain of 50% at different applied tensile strain rates (from 10 to 1000 mm min^{-1}).

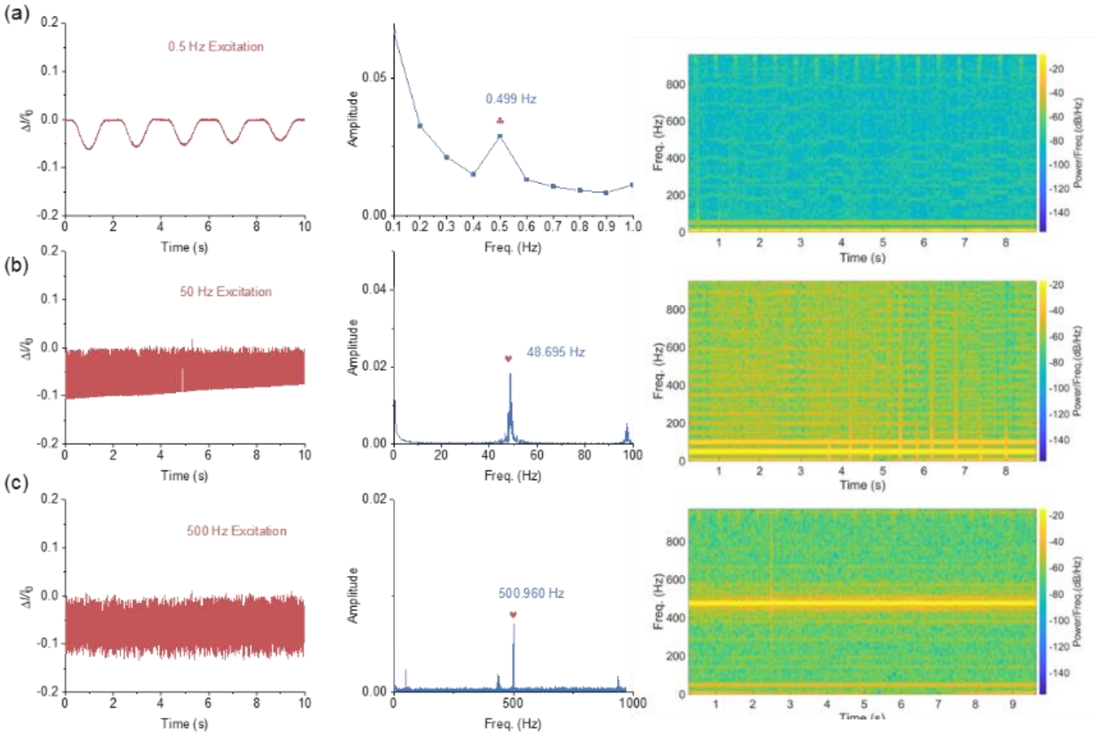

Figure S28. Current signal waveform, spectrogram, and time-frequency diagram of the TPU hybrid gel membrane under various frequencies mechanical vibration, (a)0.5 Hz, (b)50 Hz, (a)500 Hz

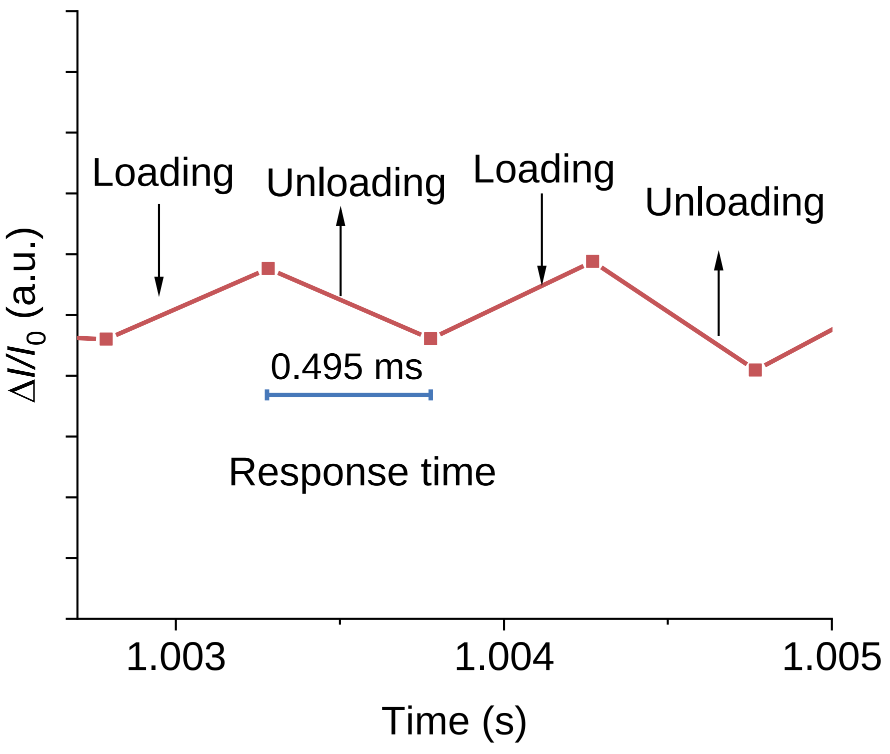

Figure S29. Response time of the TPU hybrid gel electrode under high frequency vibrations.

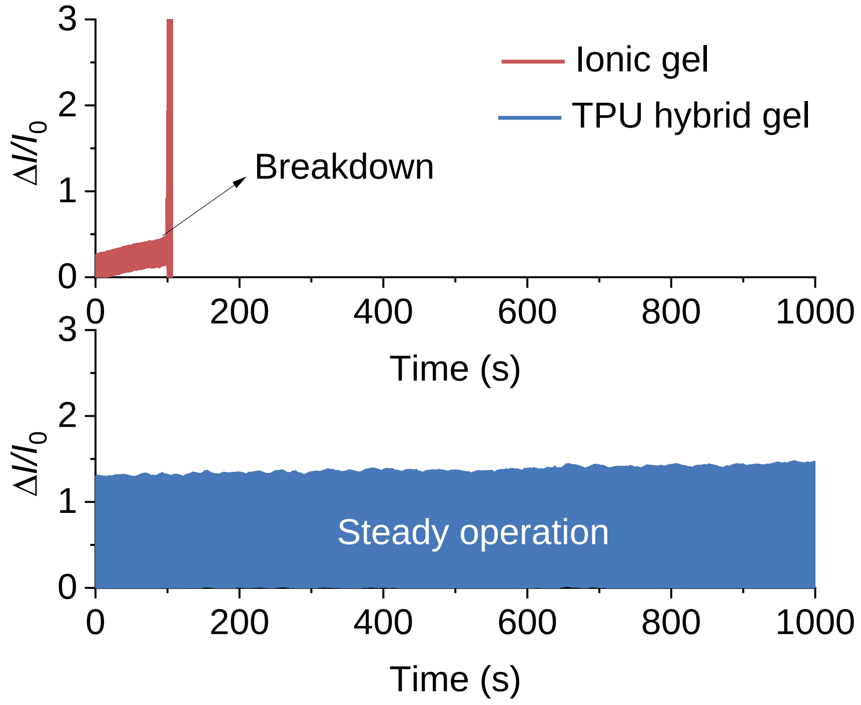

Figure S30. Cyclic performance of the notched ionic gel and TPU hybrid gel electrodes at a strain of 200%.

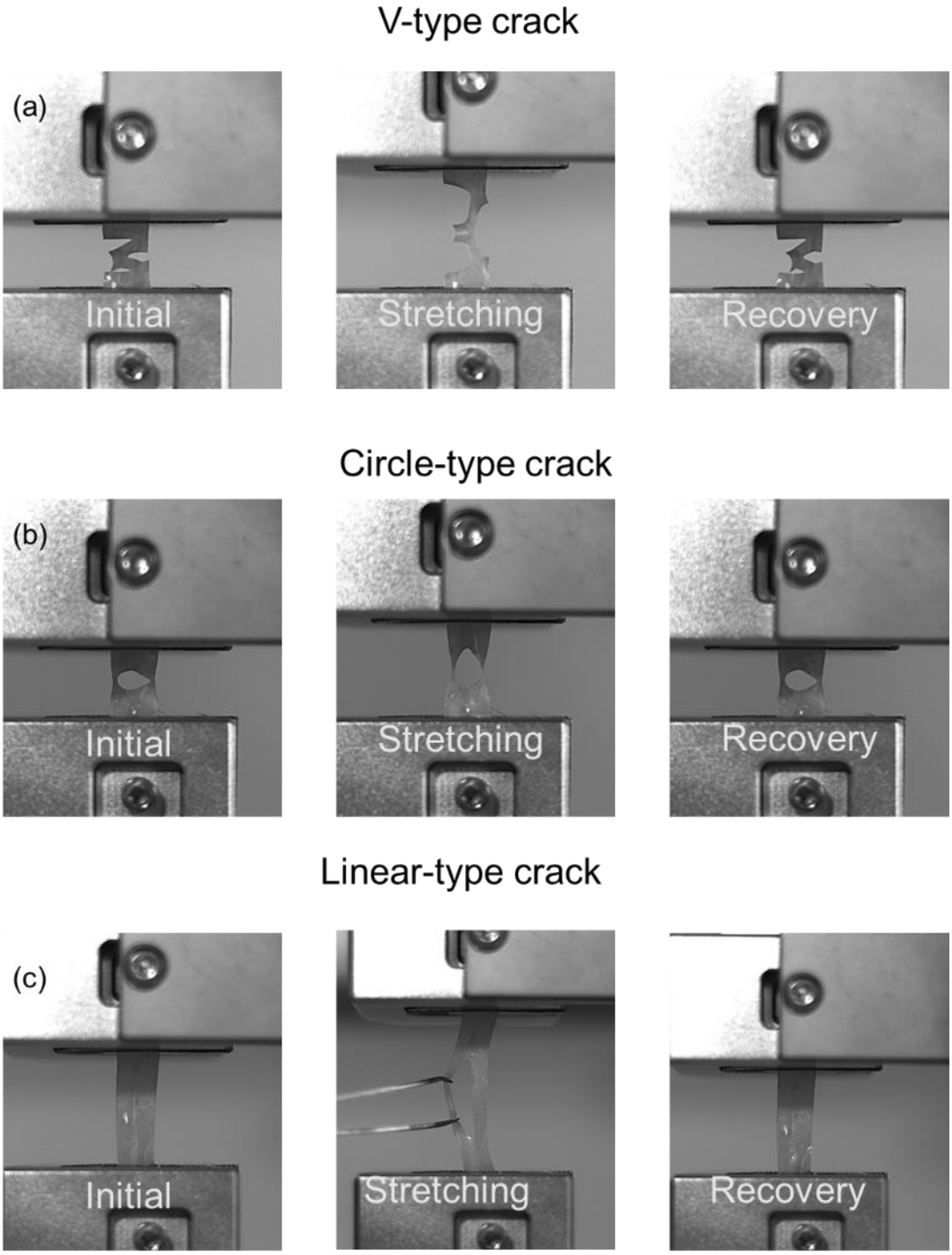

Figure S31. Photographs demonstrating the durability of notched TPU hybrid gel electrodes.

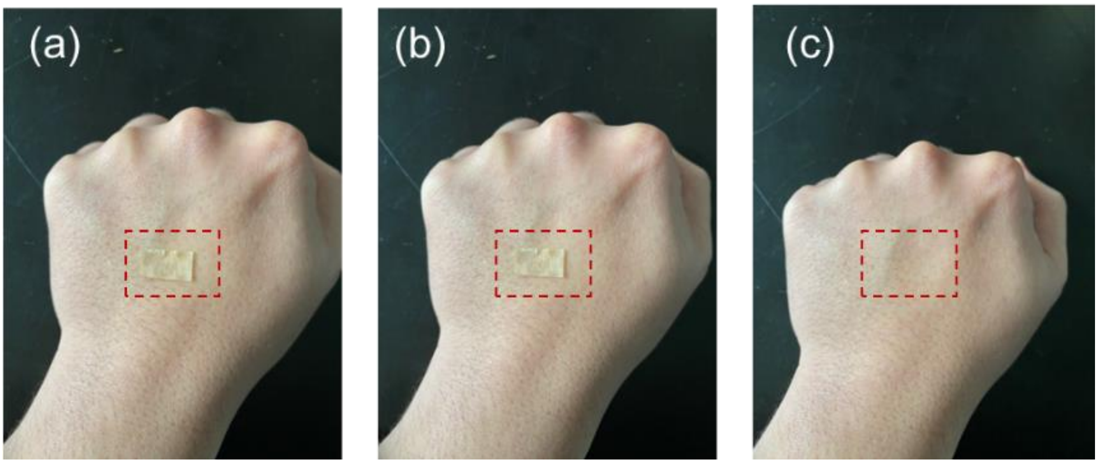

Figure S32. The TPU hybrid gel electrode was attached on the upper arm (a) and the skin surface was recorded after wearing the electrode for 10 min (b) 60 min (c)No skin irritation response was observed after wearing our electrodes for 60 min.

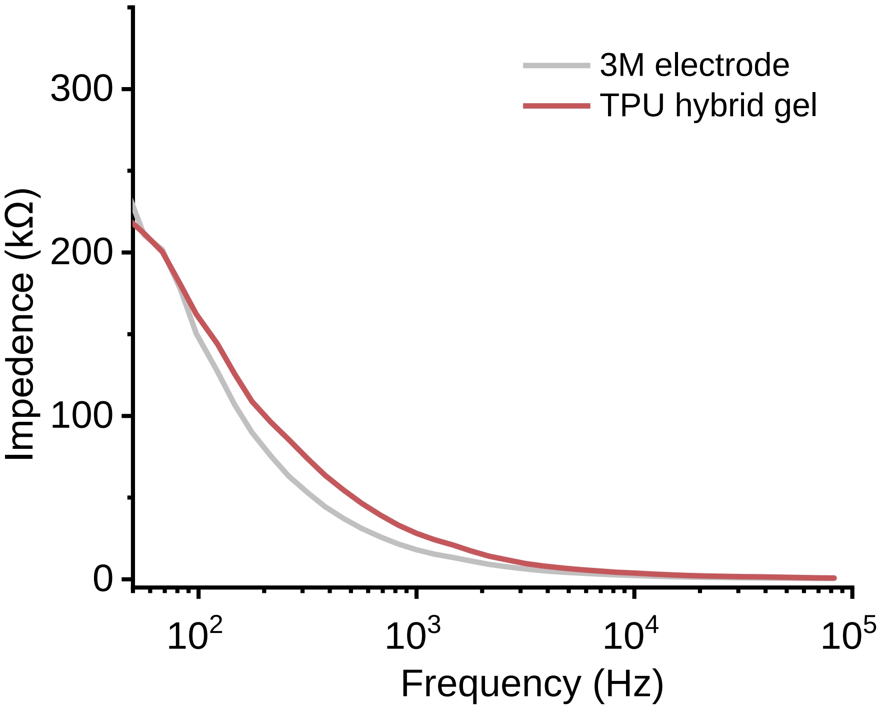

Figure S33. Comparison of impedance of the skin/TPU hybrid gel electrodes and skin/commercial 3M electrodes.

The interfacial impedance was measured by attaching pairs of electrodes with a circle shape (diameter: 20 mm), and a pitch of 40 mm on upper arm. TPU hybrid gel electrodes and commercial gel electrodes (ECG electrodes, 3M) were conducted. The interfacial impedance measurements were tested by using electrochemical workstation (ZAHNER ZENNIUM) from 10 to 10^5 Hz with an operating potential of 100 mV.

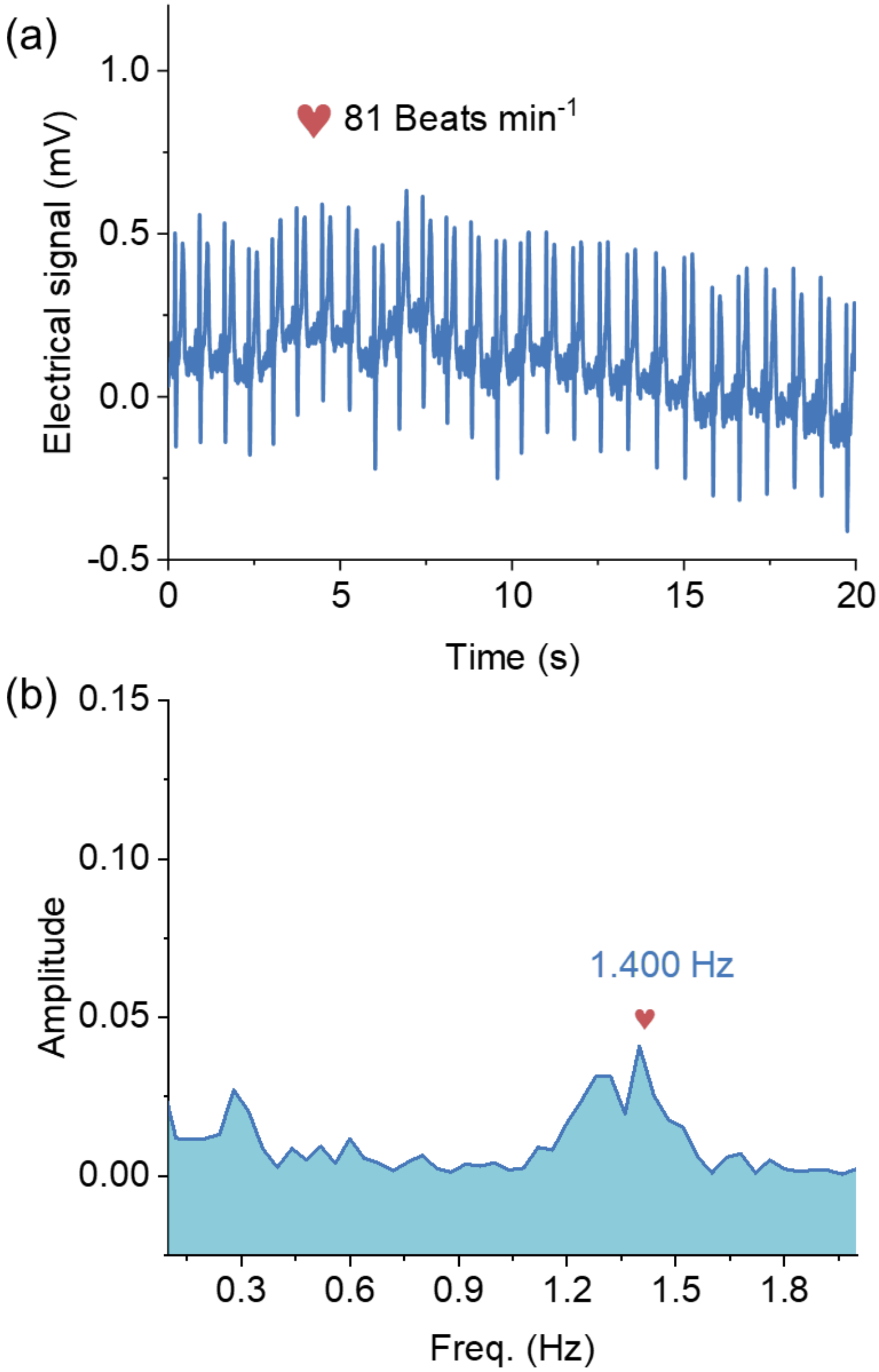

Figure S34 a) the resistance changes of the sensor in response to wrist pulse. b) Frequency of human respiration and pulse vibration

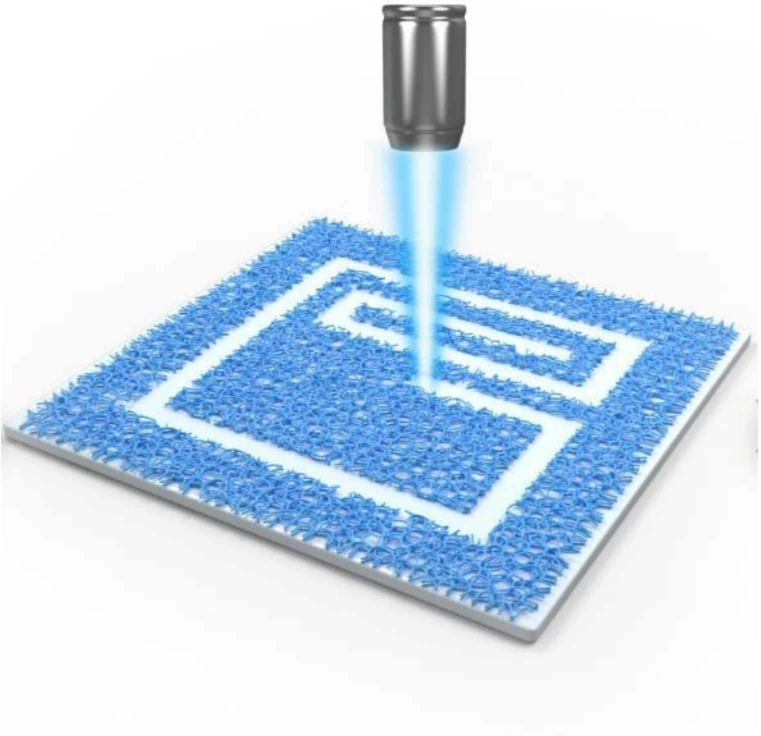

Figure S35. lithography complete the preparing of TPU based strain gauge structure.

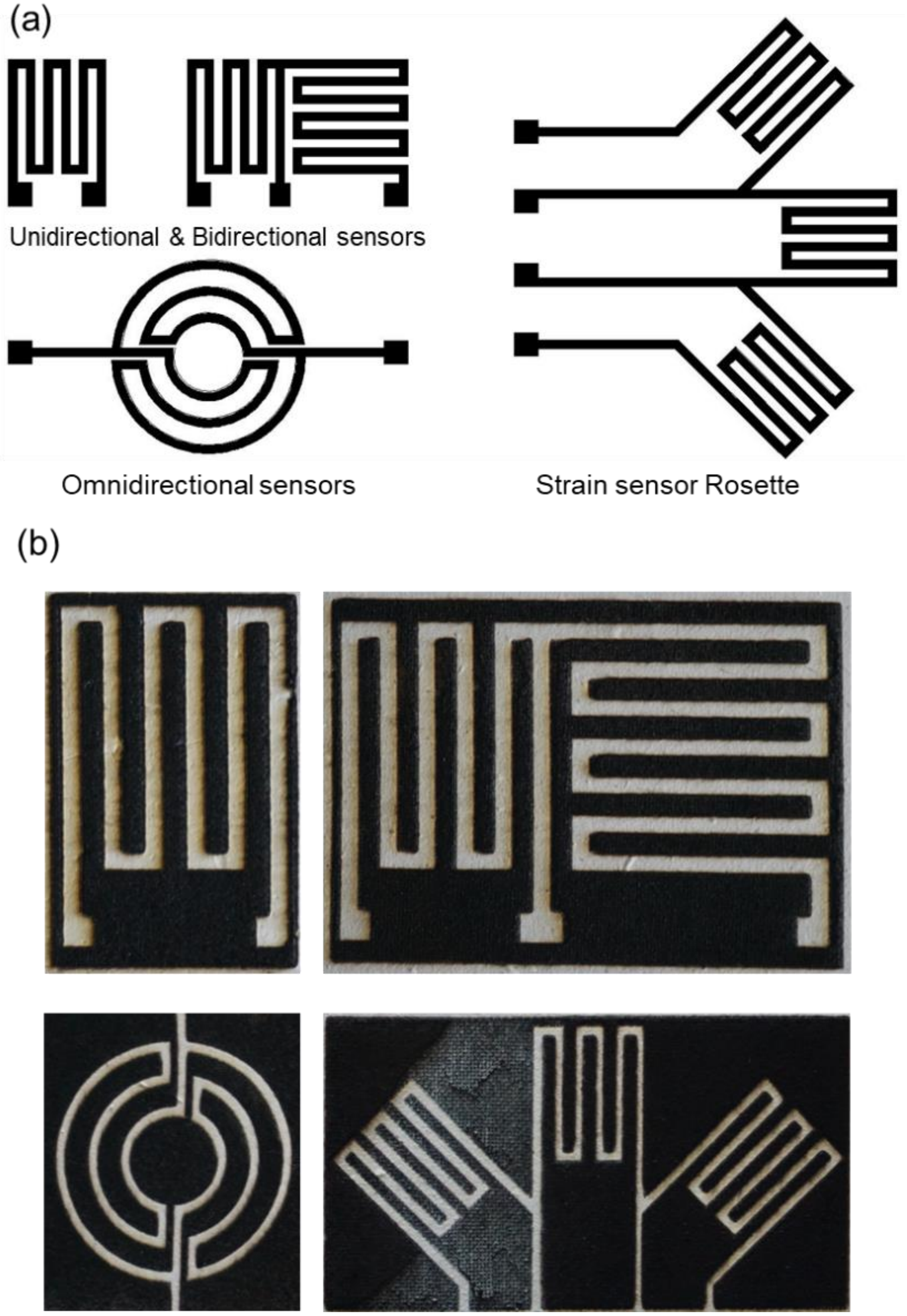

Figure S36. strain gauge sensors designed for multiple sensing scenarios.

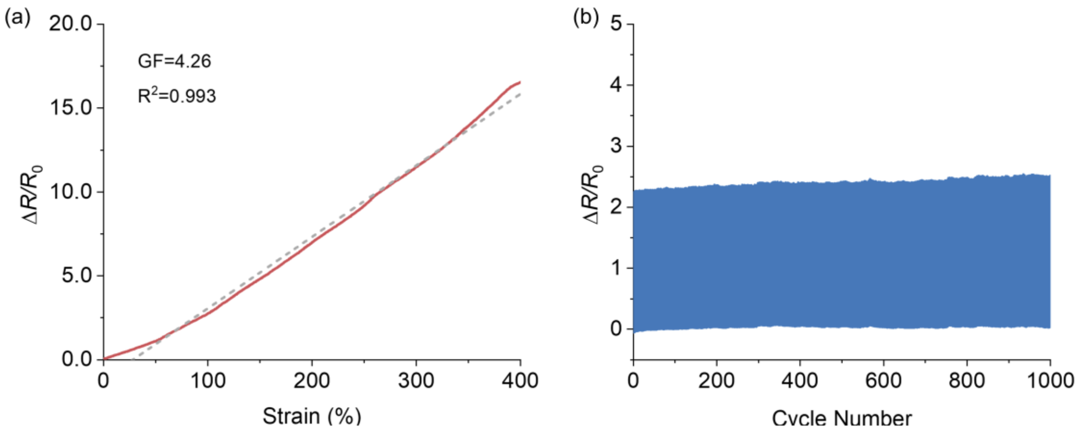

Figure S37. Electromechanical response performance of the patterned soft strain gauge. (a) Relative electrical resistance-strain response curves of the sensor. (b) The relative resistance responses of the sensor during 1000 stretching and releasing cycles at strain rates of 50%.

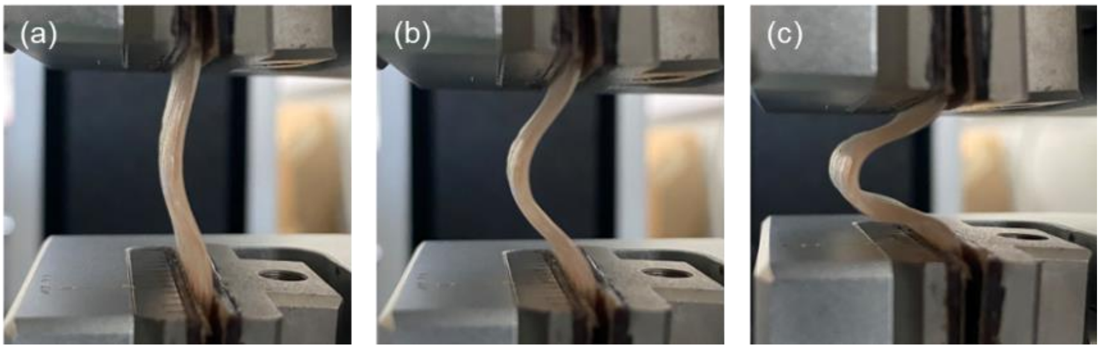

Figure S38. The photographs show of bending configurations with the strain gauge sensor.

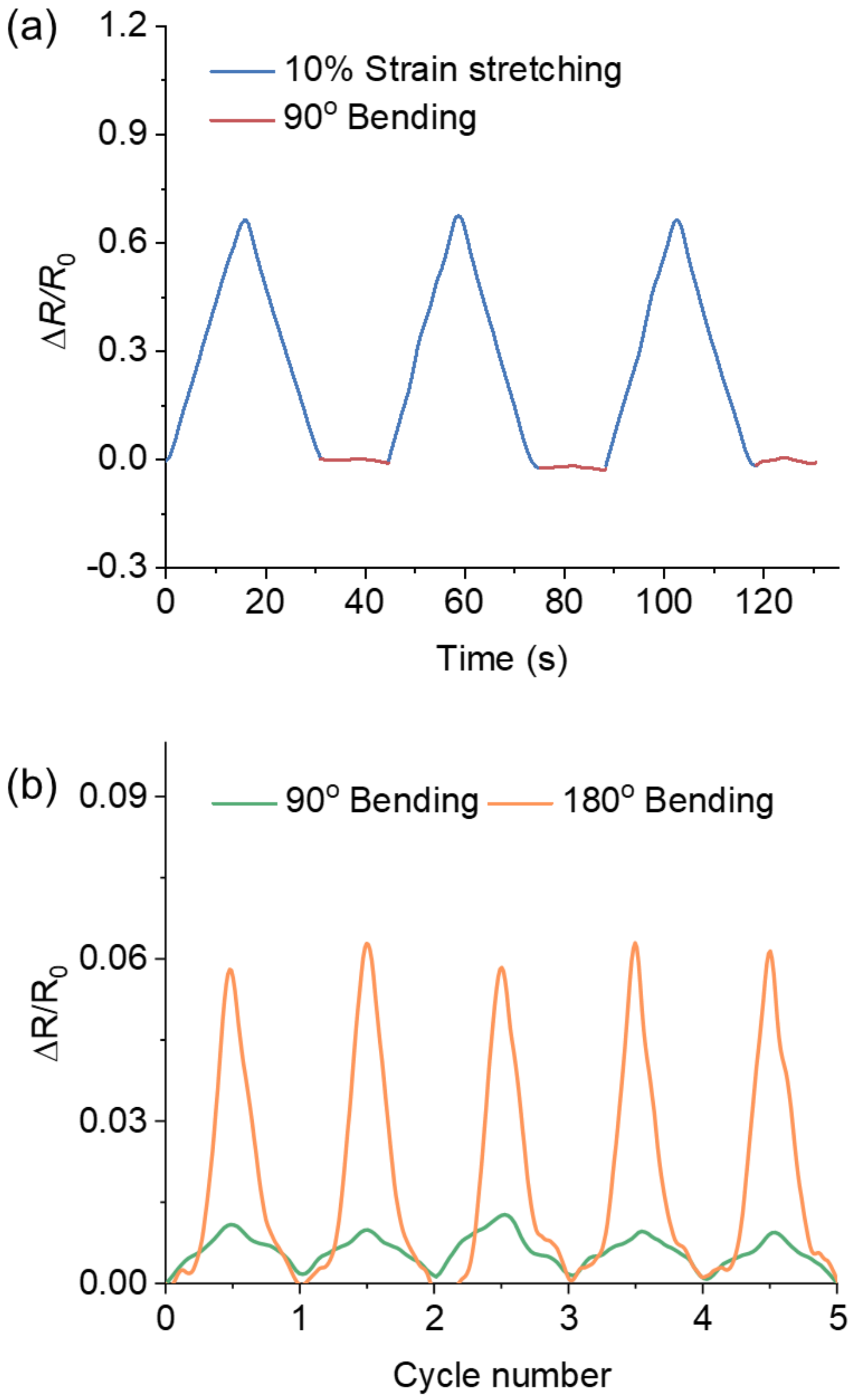

Figure S39. Strain gauge sensor response to sequential a) stretching (10% tensile strain) and bending(90°). b) stretching (10% tensile strain) and bending (90°).

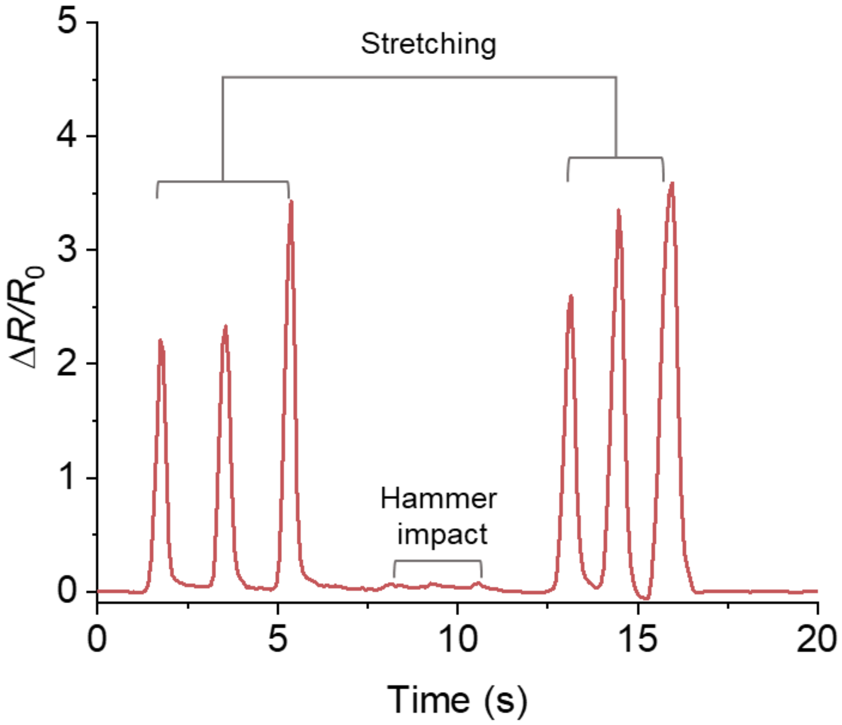

Figure S40 Demonstration of the strain gauge resilience to punctures from a strike from a hammer. The insets show a sensor sample under uniaxial stretching and a harmer strike, respectively.

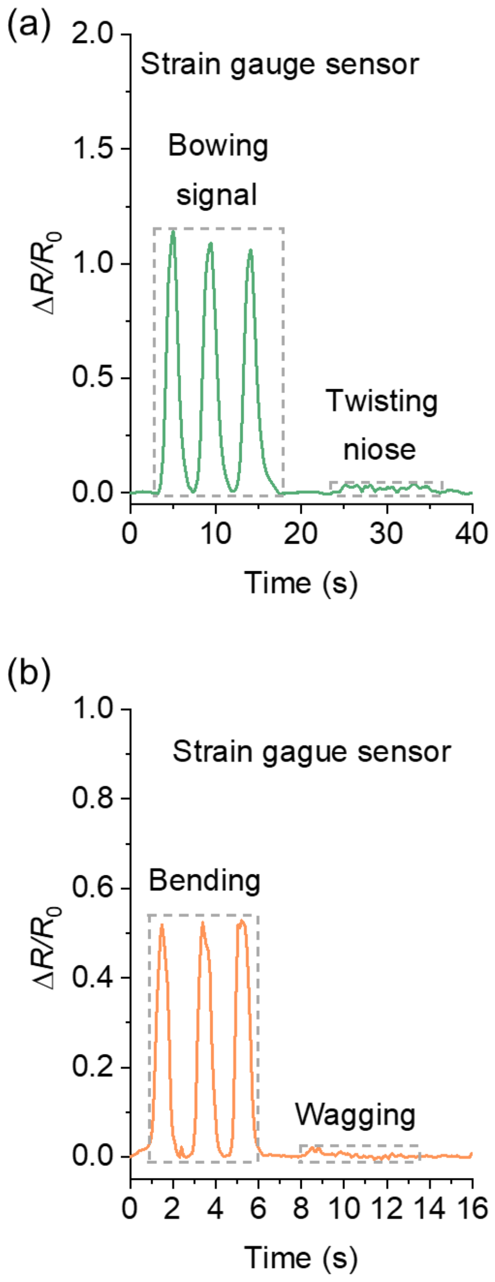

Figure S41 Signal response of TPU hybrid gel electrodes and strain gauge sensor attached on the a) back during repeated bowing/twisting motions, b) elbow during repeated bending/wagging motions.

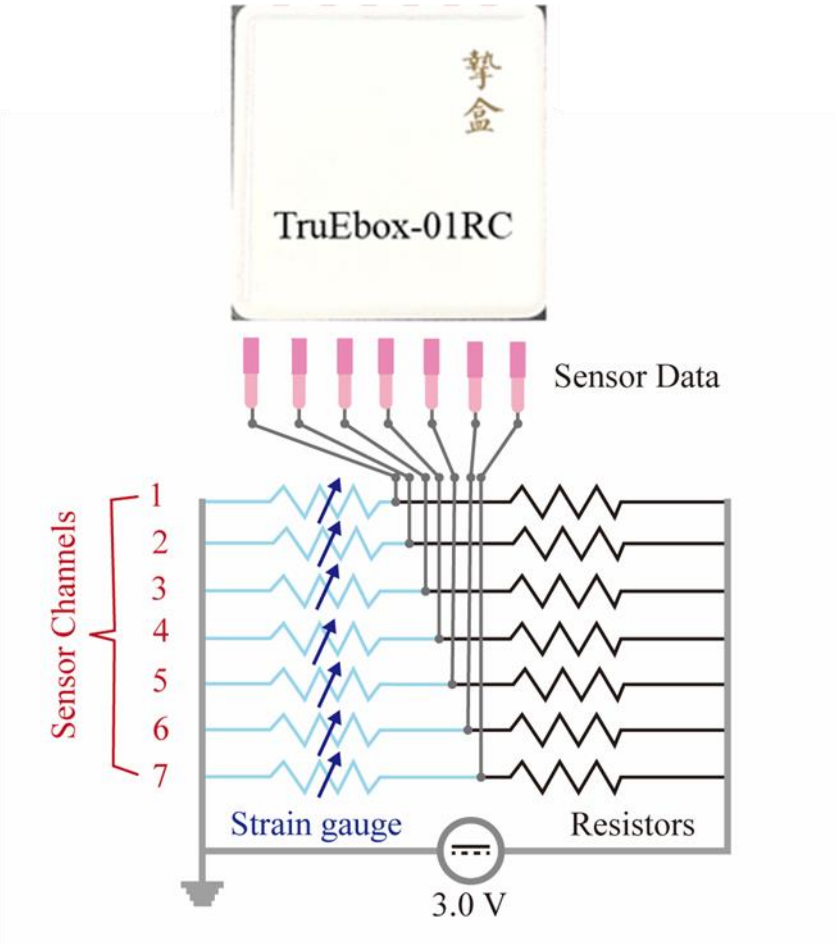

Figure S42. Equivalent circuit of a wireless sensor module.

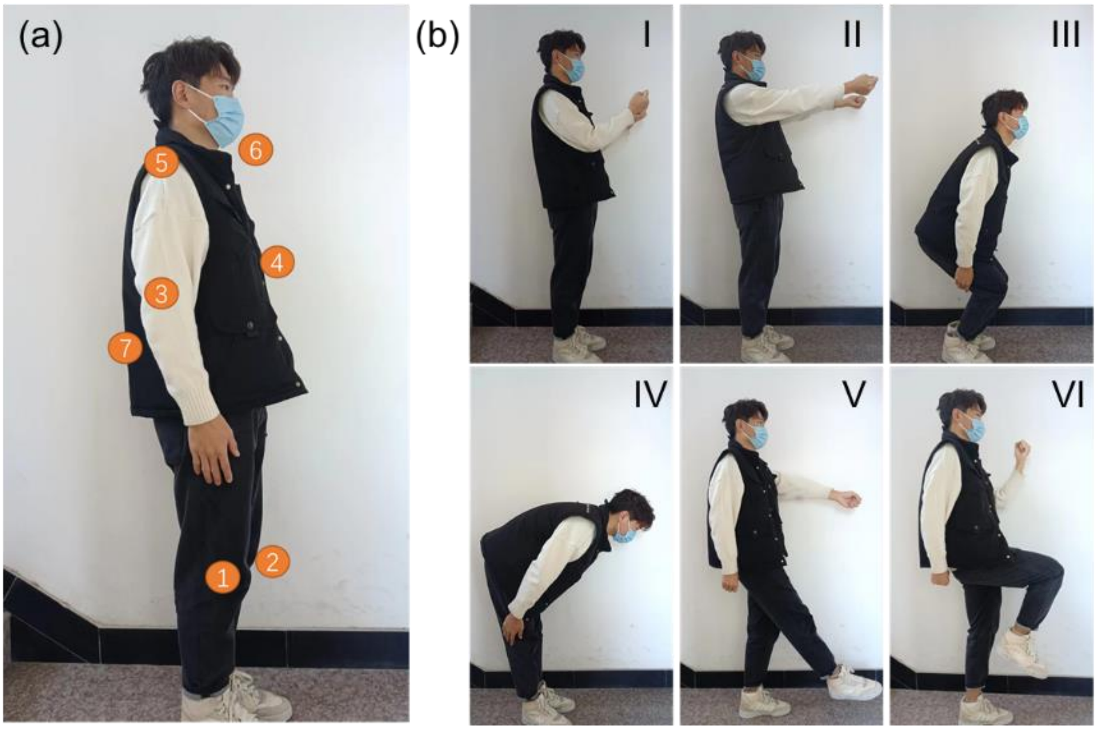

Figure S43. Images of volunteers wearing seven strain gauge sensors during different movements.

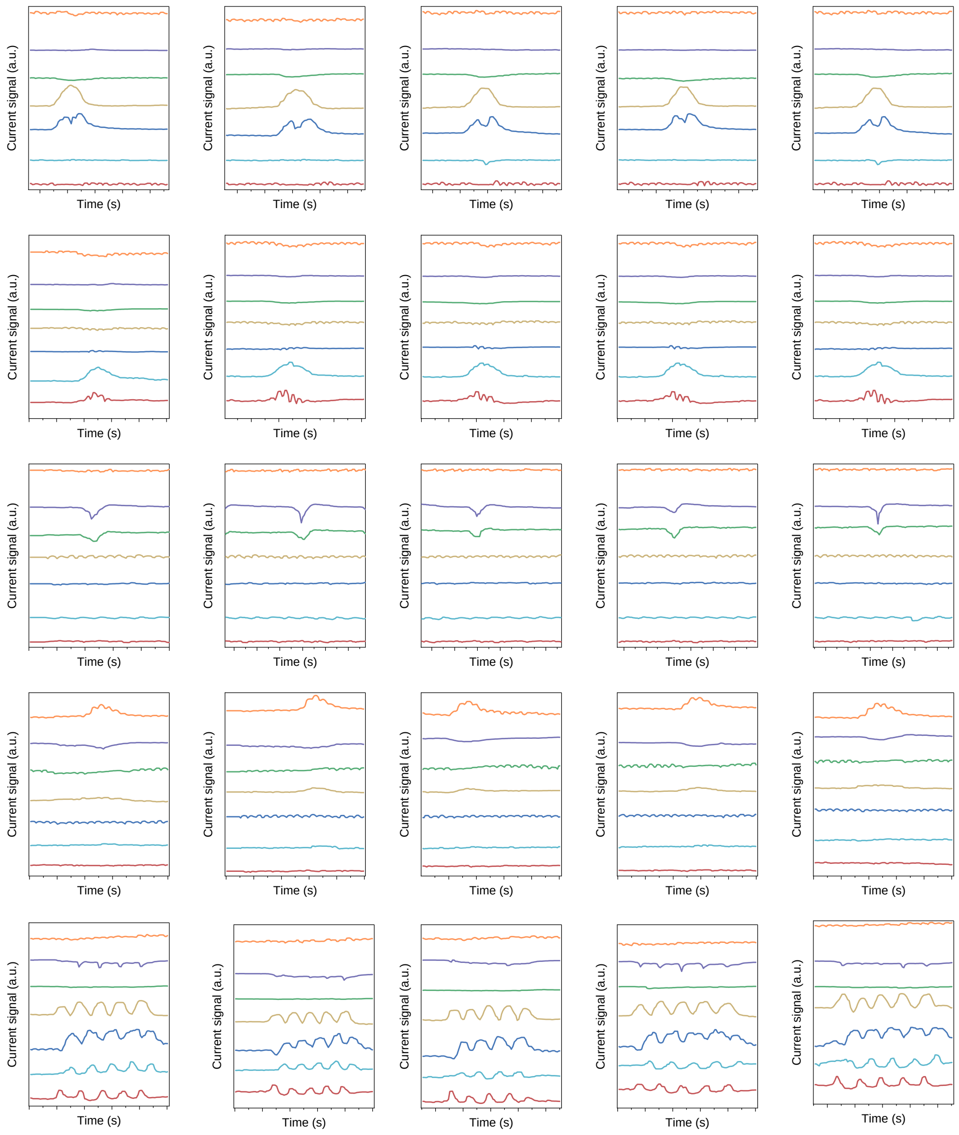

Figure S44 Some datasets used in machine learning

Supporting Video

Supporting Video S1. The TPU hybrid gel lifting a 1kg weight.

Supporting Video S2. Single-edge notch tensile test of TPU hybrid gel.

Supporting Video S3. The notched TPU hybrid gel subject to cyclic load of stretch at strain 200%.

Supporting Video S4. Vibrations measurements system.

Supporting Video S5. The durability and robustness of notched TPU hybrid gel electrodes.

Materials	Conductivit y (S m^{-1})	Strengt h (MPa)	StretStrainc h λ	Fractur e Energy (kJ m^{-2})	Fatigue threshol d (J m^{-2})	References

Supporting Video S6. TPU hybrid gel electrodes for ECG tests.

Supporting Video S7. TPU hybrid gel electrodes for EMG tests.

Table S1. EDS mapping results of TPU hybrid gel

Element	Weight %	Atomic %
C	24.45	35.44
N	14.39	17.88
O	31.97	34.79
Cl	14.86	7.30
Ca	9.62	4.18
Au	4.71	0.42

Table S2. Mechanical and electrical properties of conductive TPU hybrid gel and other polymers

TPU hybrid gel	4.38	8.4	14.25	59.6	3300	This work
Alginate/PVA/$ZnSO_4$	1.5	15	7.65	39.57	-	*Adv. Funct. Mater.2022*[2
PVA/GO hydrogel	-	8	2.25	-	1,500	*Adv. Mater.* 2022[3]
PVA/CNFF	-	7.16	1.07	1.5	-	*Adv. Mater.* 2022[4]
PVA/Sodium Citrate	-	~23.5	~29	~170	10500	*Nature 2021*[5]
Cellulose/BT hydrogel	~8.99	0.76	0.96	0.45	-	*Nat. Commun.*
PVA/ethanol/Fe^{3+}	6.5	6.5	17.1	661.54	N/A	*Adv. Funct. Mater.2022*[7
Aligned PVA	-	8.4	8	-	770	*Matter* 2021[8]
PVA/eutectogel	~0.02	20.2	5.5	42.4	-	*Adv. Funct. Mater.2022*[9
PU hybrid ionic	0.11	-	6.8	16.3	2950	*Nat. Commun.*
PHFBA-r-OEGA/LiTFSI	3.5×10^{-3}	0.77	60	22.3	-	*Adv. Funct. Mater.2021*[1
PEGA/PMMA/Li^{+}-O	-	18	300	95.265	2,682	*Nat. Commun.*
SSPU/Galinstan	-	~1	~18	111.16	-	*Nat. Commun.*
PAM/PBA-IL/CNF	~0.694	~0.349	18.1	2.65	-	*Adv. Funct. Mater.*
PTMEG/HEDS/LiTFSI	3.77×10^{-3}	27.83	27.15	-	-	*Nat. Commun.*
MEA/IBA/LiTFSI	~5.28×10^{-3}	~2.5	18.44	-	-	*Adv. Mater.* 2021[16]
TFEA/Aam/[EMIM][TFSI]	~2.92×10^{-2}	0.72	21.66	-	-	*Adv. Mater.* 2021[17]
MEA/IBA	4.27×10^{-2}	0.752	21	-	-	*Adv. Funct. Mater.*

Table S3. Electromechanical performance of TPU hybrid gel electrode and other sensing material.

Material	Linearity	Sensing range (Strain or Pressure)	Detection limit (Strain or Pressure)	Time-resolution (ms)	Durability Cycle	Reference
TPU hybrid gel	0.996	600%	0.05%	0.495	10000	This work
TPU/BNNS	-	160%	10%	-	5000	*Nat. Commun.* 2020[19]
CNT/PU/ecoflex	-	400%	0.0075%	25	10 000	*Adv. Mater.* 2022[20]
TPU/Mxene/CNT	-	70%	0.001%	5	45 000	*Adv. Mater.*

						2021[21]
TPU/CB	-	150%	0.5%	60	10000	*Nano-Micro Lett.* 2021[22]
PAM/laponite/H_3BO_3	0.996	750%	5%	10	200	*Adv. Funct. Mater.*2023[23]
TPU/CNT@MXene	-	100%	5%	-	200	*ACS Nano* 2021[24]
PDMS-Ag	-	0.65%	-	258	7000	*Adv. Mater.* 2022[25]
PEDOT:PSS-PVA	0.98	300%	0.05%	-	2000	*Adv. Mater.* 2022[26]
MXene-PVDF		50%	5%	64	20000	*Nat. Commun.* 2022[27]
PEDOT:PSS islands	0.979	100%	1%	186	1000	*Matter* 2021[28]
PDES/CMFs ICs	-	1300%	0.5%	70	1000	*Adv. Funct. Mater.* 2022[29]
MXene/Cu/PDMS	-	N/A	0.1 Pa	15	-	*Sci. Adv.* 2022[30]
CNTs-EM/Cu	-	20 kPa	2 Pa	40	10000	*Matter* 2022[31]
PS/FCNT	-	2 MPa	1.3 Pa	12	6000	*ACS Nano* 2022[32]
Mxene-CPM	0.98~0.991	1500 Pa	10 Pa	100	5000	*Matter* 2022[33]
BBP-MXene	0.998	0.2 Pa	0.0063 Pa	50	10000	*Nat. Commun.* 2022[34]

Supplementary References

[1] R. Long, C. Y. Hui, *Soft Matter* **2016**, *12*, 8069.

[2] W. Cui, Y. Zheng, R. J. Zhu, Q. F. Mu, X. Y. Wang, Z. S. Wang, S. Q. Liu, M. Li, R. Ran, *Adv. Funct. Mater.* **2022**, *32*, 2204823.

[3] X. Y. Liang, G. D. Chen, S. T. Lin, J. J. Zhang, L. Wang, P. Zhang, Y. Lan, J. Liu, *Adv. Mater.* **2022**, *34*, 2107106.

[4] H. Z. Liu, H. G. Li, Z. C. Wang, X. Wei, H. J. Zhu, M. Z. Sun, Y. Lin, L. Z. Xu, *Adv. Mater.* **2022**, *34*, 2270348.

[5] M. T. Hua, S. W. Wu, Y. F. Ma, Y. S. Zhao, Z. L. Chen, I. Frenkel, J. Strzalka, H. Zhou, X. Y. Zhu, X. M. He, *Nature* **2021**, *590*, 594.

[6] S. H. Wang, L. Yu, S. S. Wang, L. Zhang, L. Chen, X. Xu, Z. Q. Song, H. Liu, C. J. Chen, *Nat. Commun.* **2022**, *13*, 3408.

[7] X. Y. Dong, X. Guo, Q. Y. Liu, Y. J. Zhao, H. B. Qi, W. Zhai, *Adv. Funct. Mater.* **2022**, *32*, 2203610.

[8] J. H. Ni, S. T. Lin, Z. Qin, D. Veysset, X. Y. Liu, Y. C. Sun, A. J. Hsieh, R. Radovitzky, K. A. Nelson, X. H. Zhao, *Matter* **2021**, *4*, 1919.

[9] H. Zhang, N. Tang, X. Yu, M. H. Li, J. Hu, *Adv. Funct. Mater.* **2022**, *32*, 2206305.

[10] J. Q. Wang, B. H. Wu, P. Wei, S. T. Sun, P. Y. Wu, *Nat. Commun.* **2022**, *13*, 4411.

[11] P. R. Shi, Y. F. Wang, K. N. Wan, C. Zhang, T. X. Liu, *Adv. Funct. Mater.* **2022**, *32*, 2112293.

[12] M. X. Li, L. L. Chen, Y. R. Li, X. B. Dai, Z. K. Jin, Y. C. Zhang, W. W. Feng, L. T. Yan, Y. Cao, C. Wang, *Nat. Commun.* **2022**, *13*, 2279.

[13] F. Y. Sun, L. F. Liu, T. Liu, X. B. Wang, Q. Qi, Z. S. Hang, K. Chen, J. H. Xu, J. J. Fu, *Nat. Commun.* **2023**, *14*, 130.

[14] X. Yao, S. F. Zhang, L. W. Qian, N. Wei, V. Nica, S. Coseri, F. Han, *Adv. Funct. Mater.* **2022**, *32*, 2204565.

[15] J. Chen, Y. Y. Gao, L. Shi, W. Yu, Z. J. Sun, Y. F. Zhou, S. Liu, H. Mao, D. Y. Zhang, T. Q. Lu, Q. Chen, D. M. Yu, S. J. Ding, *Nat. Commun.* **2022**, *13*, 4868.

[16] B. R. B. Yiming, Y. Han, Z. L. Han, X. N. Zhang, Y. Li, W. Z. Lian, M. Q. Zhang, J. Yin, T. L. Sun, Z. L. Wu, T. F. Li, J. Z. Fu, Z. Jia, S. X. Qu, *Adv. Mater.* **2021**, *33*, 6111.

[17] L. G. Xu, Z. K. Huang, Z. S. Deng, Z. K. Du, T. L. Sun, Z. H. Guo, K. Yue, *Adv. Mater.* **2021**, *33*, 2105306.

[18] B. R. B. Yiming, X. Guo, N. Ali, N. Zhang, X. N. Zhang, Z. L. Han, Y. C. Lu, Z. L. Wu, X. L. Fan, Z. Jia, S. X. Qu, *Adv. Funct. Mater.* **2021**, *31*, 2102773.

[19] C. X. Tan, Z. G. Dong, Y. H. Li, H. G. Zhao, X. Y. Huang, Z. C. Zhou, J. W. Jiang, Y. Z. Long, P. K. Jiang, T. Y. Zhang, B. Sun, *Nat. Commun.* **2020**, *11*, 3530.

[20] J. W. Gao, Y. B. Fan, Q. T. Zhang, L. Luo, X. Q. Hu, Y. Li, J. C. Song, H. J. Jiang, X. Y. Gao, L. Zheng, W. Zhao, Z. H. Wang, W. Ai, Y. Wei, Q. B. Lu, M. Z. Xu, Y. T. Wang, W. T. Song, X. W. Wang, W. Huang, *Adv. Mater.* **2022**, *34*, 2107511.

[21] Y. Zhao, W. C. Gao, K. Dai, S. Wang, Z. Q. Yuan, J. N. Li, W. Zhai, G. Q. Zheng, C. F. Pan, C. T. Liu, C. Y. Shen, *Adv. Mater.* **2021**, *33*, 2102332.

[22] X. Wang, X. H. Liu, D. W. Schubert, *Nanomicro Lett* **2021**, *13*, 64.

[23] J. Zou, X. Jing, Z. Chen, S. J. Wang, X. S. Hu, P. Y. Feng, Y. J. Liu, *Adv. Funct. Mater.* **2023**, 2213895.

[24] H. C. Wang, R. C. Zhou, D. H. Li, L. R. Zhang, G. Z. Ren, L. Wang, J. H. Liu, D. Y. Wang, Z. H. Tang, G. Lu, G. Z. Sun, H. D. Yu, W. Huang, *ACS Nano* **2021**, *15*, 9690.

[25] L. P. Liu, S. C. Niu, J. Q. Zhang, Z. Z. Mu, J. Li, B. Li, X. C. Meng, C. C. Zhang, Y. Q. Wang, T. Hou, Z. W. Han, S. Yang, L. Q. Ren, *Adv. Mater.* **2022**, *34*, 2200823.

[26] Z. Q. Shen, Z. L. Zhang, N. B. Zhang, J. H. Li, P. W. Zhou, F. Q. Hu, Y. Rong, B. Y. Lu, G. Y. Gu, *Adv. Mater.* **2022**, *34*, 2203650.

[27] H. T. Yang, J. L. Li, X. Xiao, J. H. Wang, Y. F. Li, K. R. Li, Z. P. Li, H. C. Yang, Q. Wang, J. Yang, J. S. Ho, P. L. Yeh, K. Mouthaan, X. N. Wang, S. Shah, P. Y. Chen, *Nat. Commun.* **2022**, *13*, 5311.

[28] H. Liu, S. M. Zhang, Z. K. Li, T. J. Lu, H. S. Lin, Y. Z. Zhu, S. Ahadian, S. Emaminejad, M. R. Dokmeci, F. Xu, A. Khademhosseini, *Matter* **2021**, *4*, 2886.
[29] X. Sun, Y. L. Zhu, J. Y. Zhu, K. Le, P. Servati, F. Jiang, *Adv. Funct. Mater.* **2022**, *32*, 2202533.
[30] G. Y. Gou, X. S. Li, J. M. Jian, H. Tian, F. Wu, J. Ren, X. S. Geng, J. D. Xu, Y. C. Qiao, Z. Y. Yan, G. Dun, C. W. Ahn, Y. Yang, T. L. Ren, *Sci. Adv.* **2022**, *8*, eabn2156.
[31] X. Wei, H. Li, W. J. Yue, S. Gao, Z. X. Chen, Y. Li, G. Z. Shen, *Matter* **2022**, *5*, 1481.
[32] Y. X. Hou, L. Wang, R. Sun, Y. A. Zhang, M. X. Gu, Y. H. Zhu, Y. B. Tong, X. Y. Liu, Z. X. Wang, J. Xia, Y. G. Hu, L. Wei, C. L. Yang, M. Chen, *Acs Nano* **2022**, *16*, 8358.
[33] X. L. Shi, Y. B. Zhu, X. Q. Fan, H. A. Wu, P. Q. Wu, X. Y. Ji, Y. S. Chen, J. J. Liang, *Matter* **2022**, *5*, 1547.
[34] X. L. Shi, X. Q. Fan, Y. B. Zhu, Y. Liu, P. Q. Wu, R. H. Jiang, B. Wu, H. A. Wu, H. Zheng, J. B. Wang, X. Y. Ji, Y. S. Chen, J. J. Liang, *Nat. Commun.* **2022**, *13*, 1119.
